# Synthesis and Application of 1,2-Aminoalcohols with Neoisopulegol-Based Octahydrobenzofuran Core

**DOI:** 10.3390/molecules25010021

**Published:** 2019-12-19

**Authors:** Fatima Zahra Bamou, Tam Minh Le, Bettina Volford, András Szekeres, Zsolt Szakonyi

**Affiliations:** 1 Institute of Pharmaceutical Chemistry, University of Szeged, Interdisciplinary excellent center, H-6720 Szeged, Eötvös utca 6, Hungary; fatima.z@pharm.u-szeged.hu (F.Z.B.); leminhtam@pharm.u-szeged.hu (T.M.L.); 2 MTA-SZTE Stereochemistry Research Group, Hungarian Academy of Sciences, H-6720 Szeged, Eötvös utca 6, Hungary; 3 Department of Microbiology, University of Szeged, 6726 Szeged, Közép fasor 52, Hungary; bettina.volford86@gmail.com (B.V.); andras.j.szekeres@gmail.com (A.S.); 4 Interdisciplinary Centre of Natural Products, University of Szeged, H-6720 Szeged, Eötvös utca 6, Hungary

**Keywords:** neoisopulegol, octahydrobenzofuran, 1,2-aminoalcohol, chiral catalyst, antimicrobial activity

## Abstract

A library of 1,2-aminoalcohol derivatives with a neoisopulegol-based octahydrobenzofuran core was developed and applied as chiral catalysts in the addition of diethylzinc to benzaldehyde. The allylic chlorination of (+)-neoisopulegol, derived from natural (–)-isopulegol followed by cyclization, gave the key methyleneoctahydrobenzofuran intermediate. The stereoselective epoxidation of the key intermediate and subsequent oxirane ring opening with primary amines afforded the required 1,2-aminoalcohols. The ring closure of the secondary amine analogues with formaldehyde provided spiro-oxazolidine ring systems. The dihydroxylation of the methylenetetrahydrofuran moiety with OsO_4_/NMO (4-methylmorpholine *N*-oxide) resulted in the formation of a neoisopulegol-based diol in a highly stereoselective reaction. The antimicrobial activity of both the aminoalcohol derivatives and the diol was also explored.

## 1. Introduction

The benzofuran moiety is prevalent in a great number of biologically active compounds and natural products [1,2]. Moreover, compounds bearing this ring system are also promising key intermediates in the preparation of natural products and clinical medicines [3,4,5]. Due to the availability of these building blocks, numerous methods have been developed for the preparation of benzofuran systems [6,7,8,9,10,11]. However, only a few examples of the synthesis of octahydrobenzofuran derivatives have been reported including free-radical reactions [12,13], hydrogenation [14,15], tandem conjugate addition [16], base- [17] or acid-catalyzed cyclization [18], and photochemical rearrangement [19]. Furthermore, octabenzohydrofuran derivatives are well-known versatile precursors for the construction of a variety of therapeutic drugs [20]. For example, (±)-adunctin B and its modified derivatives that bear a hexahydrobenzofurane moiety have shown antibacterial effects toward *Micrococcus luteus* [21]. (–)-Siccanin exhibits potent antifungal activity against several pathogenic fungi, and its clinical effectiveness against surface mycosis is also known [22].

The 1,2-aminoalcohol moiety is present in a wide range of compounds that exhibit pharmaceutically and biologically interesting properties [23]. For example, compounds bearing the hydroxyethylamine core have the capacity to inhibit aspartic protease enzymes and are widely used as anti-HIV [24,25], antimalarial [26,27,28], and antileishmanial [29] agents. The 1,2-aminoalcohol function is found in a broad range of *β*-adrenergic blockers that are used extensively in the management of cardiovascular disorders [30], including hypertension, angina pectoris and cardiac arrhythmias, and other disorders that are related to the sympathetic nervous system [31,32].

1,2-aminoalcohols have also been demonstrated to be excellent chiral auxiliaries and chiral catalysts in asymmetric synthesis [33]. To achieve new, efficient, and commercially available chiral catalysts, natural chiral terpenes, such as *α*-pinene [34,35,36,37,38], *β*-pinene [34,39], (–)-3-carene [39,40], (–)-verbenone [41,42], (–)-fenchone [43,44], (+)-camphor [43,45,46], and (–)-menthone [47] have proven to be excellent sources for the synthesis of bifunctional chiral compounds and heterocycles.

In the present work, we set out to create a compound library with a (+)-neoisopulegol-based octahydrobenzofuran core and 1,2-aminoalcohol moieties. The synthesis started from commercially available (–)-isopulegol and then utilizing the resulting 1,2-aminoalcohol derivatives as chiral catalysts in the enantioselective addition of diethylzinc to benzaldehyde. Furthermore, the antimicrobial activities of the synthesized compounds were also tested on multiple bacterial and fungal strains.

## 2. Results

### 2.1. Synthesis of Key Intermediate **3**

**Figure 1:**
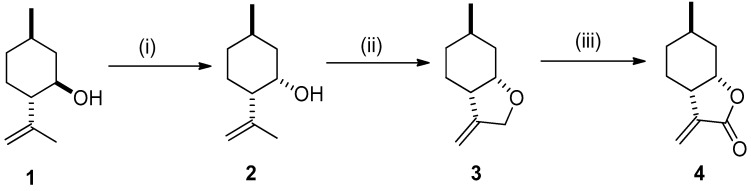
Synthesis of (–)-isopulegol-based methylenetetrahydrofuran **3**: (**i**) PCC (2 equivalents), DCM (Dichloromethane), 25 °C, 48 h, 80% than L-selectride (1.5 equivalents) dry THF, −78 °C, 1 h, 90% [48,49,50,51]; (**ii**) Ca(OCl)_2_, DCM, rt 25 °C, 3 h, 70% than NaH (2 equivalents), dry THF, 50 °C, 6 h [52,53,54,55]; (**iii**) CrO_3_ (3 equivalents), DCM/pyridine, reflux, 1.5 h, 84% [55,56].

Key intermediate (–)-3-methylenetetrahydrofuran **3** was prepared from commercially available (–)-isopulegol **1** by oxidizing its hydroxyl function, followed by the stereoselective reduction of the resulting carbonyl group, thus providing (+)-neoisopulegol **2** [48,49,50,51]. The allylic chlorination of (+)-neoisopulegol **2** was followed by the cyclization-produced (–)-methylenetetrahydrofuran **3** [52,53,54,55], which was transformed into (−)-methylenetetrahydrofuran **4** by allylic oxidation after applying the literature method [55,56] (Figure 1).

### 2.2. Synthesis of Ispulegol-Based 1,2-Aminoalcohols

**Figure 2:**
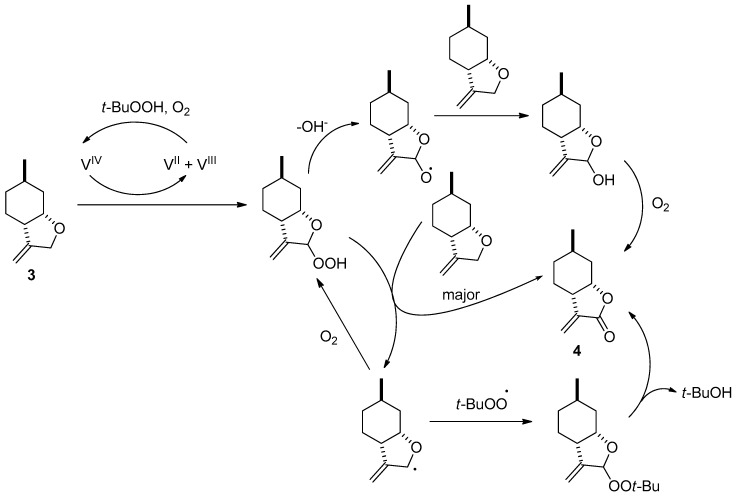
Proposed reaction pathway of allylic oxidation of **3**.

**Scheme 1:**
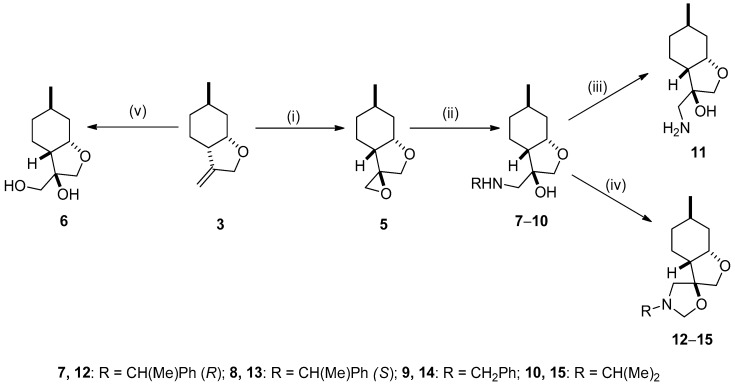
(**i**) mCPBA (2 equivalents), Na_2_HPO_4_. 2H_2_O (3 equivalents), 25 °C, 2 h, 23%; (**ii**) RNH_2_ (2 equivalents), LiClO_4_ (1 equivalent), MeCN, 70–80 °C, 6 h, 65–85%; (**iii**) 5% Pd/C, H_2_ (1 atm), MeOH, 25 °C, 24 h, 70–75%; (**iv**) 35% HCHO, Et_2_O, 25 °C, 1 h, 50–90%; (**v**) 2% OsO_4_/*t*-BuOH, 50% NMO/H_2_O, acetone, 25 °C, 24 h, 50%.

Our previous work has shown that epoxidation with *t*-BuOOH in the presence of vanadyl acetylacetonate (VO(acac)_2_) as a catalyst can be successfully applied to prepare a new family of neoisopulegol-based chiral aminodiol libraries [57]. However, upon applying this condition with **3**, (–)-α-methylene-γ-butyrolactone **4** was observed as the major product. The formation of **4** was explained by the allylic oxidation process shown in Figure 2 [58,59]. Finally, the synthesis of epoxide **5** was achieved by reacting **3** with mCPBA (*meta*-Chloroperoxybenzoic acid) in a stereoselective reaction (Scheme 1) [60,61,62,63].

Given that we clearly demonstrated in previous works [64,65] that the substitution of the nitrogen atom of aminoalcohols definitely influences the efficiency of their catalytic activity, aminoalcohol library **7**–**10** was prepared through the aminolysis of epoxide **5** with primary amines and lithium perchlorate as a catalyst [66,67]. Since the ring closure of monoterpene-based aminoalcohols with rigid structures has been shown to enhance their catalytic potential in our earlier experiments [64,65,68], the treatment of aminoalcohols **7**–**10** with formaldehyde at room temperature resulted in the formation of spiro-oxazolidines **12**–**15**. The debenzylation by hydrogenolysis of compounds **7**–**9** over Pd/C in MeOH provided primary aminoalcohol **11** in moderate yields (Scheme 1). Since neither the hydrogenolysis of *N*-benzyl analogues **7**–**10** nor the formation of an oxazolidine ring system by ring closure with formaldehyde had an effect on the absolute configuration of C-3, the relative configuration of the chiral centers of **11**–**15** is known to be the same as that of **7**–**10** [64,65,68].

The syn-selective dihydroxylation of compound **3** with OsO_4_ in the presence of a stoichiometric amount of the co-oxidant, NMO (4-methylmorpholine *N*-oxide) produced product **6** as a single diastereomer in a moderate yield [68,69] (Scheme 1).

**Figure 3:**
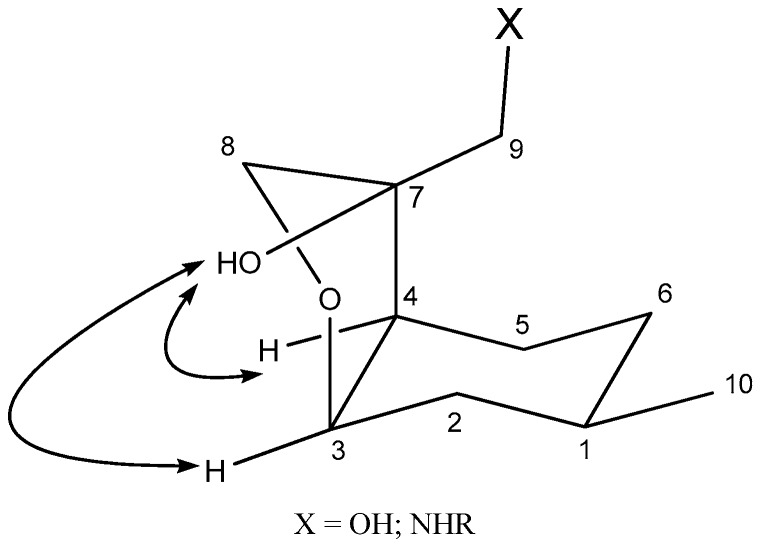
Determination of relative configuration of aminoalcohols **7**–**10** and diol **6**.

The relative stereochemistry of aminoalcohols **7**–**10** and diol **6** was established by coupling constant data and the NOESY (Nuclear Overhauser Effect SpecroscopY) spectral analysis. The large coupling constant of H-9 (*J*_4,9_ = 11.2 Hz with **6** and *J*_4,9_ = 12.2 Hz with **7**–**10**) indicated that it should be axially oriented, while the coupling constant values between H-3 and H-4 (*J*_4,3_ = *J*_3,4_ = 2.3 Hz with **6** and *J*_4,3_ = *J*_3,4_ = 2.2–3.0 Hz with **7**–**10**) supported their equatorial orientation. Furthermore, NOESY correlations between OH-7 and H-3 as well as OH-7 and H-4 protons in DMSO-*d*6 (Dimethylsulfoxide-*d*6) indicated that these groups were oriented in the same direction (see Supporting Information), Therefore, the structures of **6**–**10** were concluded, as shown on Figure 3. The stereochemistry of **11** and **12**–**15** was proven in a similar manner by 1D and 2D NMR measurements.

### 2.3. Application of Aminoalcohol Derivatives as Chiral Ligands for Catalytic Addition of Diethylzinc to Benzaldehyde

**Scheme 2:**
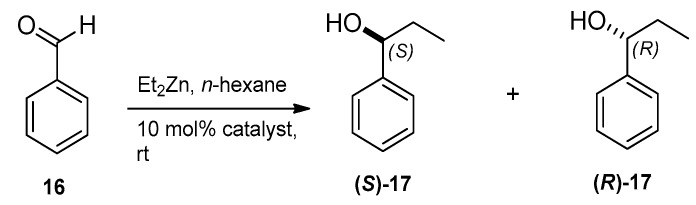
Model reaction for enantioselective catalysis.

Aminoalcohol derivatives **7**–**15** were applied as chiral catalysts in the enantioselective addition of diethylzinc to benzaldehyde **16** to form (*S*)- and (*R*)-1-phenyl-1-propanol **17** (Scheme 2).

The enantiomeric purity of 1-phenyl-1-propanols (*S*)-**17** and (*R*)-**17** was determined by GC analysis on a Chirasil-DEX CB column using literature methods [70,71]. A low-to-moderate enantioselectivity was observed. Aminoalcohols afforded the (S)-enantiomer (except **7**, where a weak (*R*) selectivity was observed), while the formation of the (*R*)-enantiomer was predominant when spiro-oxazolidines were applied as catalysts (Table 1). Aminoalcohol **8** showed the best catalytic activity (*ee* = 40%) with an (*S*)-selectivity (entry 2). The obtained results clearly indicate that the spiro-oxazolidine ring had a poorer catalytic performance, probably due to the flexible spiro system. These results are in good correlation with those observed with pinane- or sabinane-based spiro-oxazolidines in our earlier studies [72,73].

### 2.4. Antimicrobial Effects

Since several aminoalcohols have been shown to exert antimicrobial activities on various bacterial and fungal strains [74,75], the antimicrobial activities of the prepared aminoalcohol analogues and diol **6** were tested against two yeasts, as well as two Gram-positive and two Gram-negative bacteria (Table 2). Compounds **8** and **12** inhibited the studied Gram-positive bacteria with efficiencies over 20%, while other derivatives showed weak activities. In the case of *Bacillus subtilis*, **8** showed more potential antimicrobial activity, while for *Staphylococcus aureus*, **12** proved to be the most effective agent. Furthermore, only **9** showed an inhibition activity over 30% for *Pseudomonas aeruginosa,* while it had only a moderate effect against *Escherichia coli*. All compounds presented low-to-moderate inhibitions against *E. coli* in the range of 5–30%.

According to our results, *N*-substituted 1,2-aminoalcohols **7**–**10** had a moderate activity against both Gram-negative and Gram-positive bacteria. Most of the ring-closing oxazolidine products (**12**–**14**) showed a similar moderate antibacterial activity. The removal of the nitrogen substituent of the aminoalcohols led to the loss of antibacterial activity (see amino diol **6**). None of the aminoalcohol derivatives exhibited any remarkable antifungal effect, while diol **6** showed significant antifungal activity against *Candida krusei* (Table 2).

## 3. Materials and Methods

### 3.1. Materials and General Methods

Commercially available compounds were used as-obtained from suppliers (Molar Chemicals Ltd., Halásztelek, Hungary; Merck Ltd., Budapest, Hungary and VWR International Ltd., Debrecen, Hungary), while solvents were dried according to standard procedures. Optical rotations were measured in MeOH at 20 °C with a PerkinElmer 341 polarimeter (PerkinElmer Inc., Shelton, CT, USA). Chromatographic separations and monitoring of reactions were carried out on a Merck Kieselgel 60 (Merck Ltd., Budapest, Hungary). Elemental analyses of all compounds were performed on a PerkinElmer 2400 Elemental Analyzer (PerkinElmer Inc., Waltham, MA, USA). GC measurements for the direct separation of commercially available enantiomers of isopulegol to determine the enantiomeric purity of starting material **1** and the separation of *O*-acetyl derivatives of enantiomers were performed on a Chirasil-DEX CB column (2500 × 0.25 mm I.D.) on a PerkinElmer Autosystem XL GC consisting of a flame ionization detector (PerkinElmer Corporation, Norwalk, CT, USA) and a Turbochrom Workstation data system (PerkinElmer Corp., Norwalk, CT, USA). Melting points were determined on a Kofler apparatus (Nagema, Dresden, Germany) and were uncorrected. ^1^H- and ^13^C-NMR spectra were recorded on a Brucker Avance DRX 500 spectrometer [500 MHz (^1^H) and 125 MHz (^13^C), δ = 0 (TMS, Tetramethylsilane)]. Chemical shifts are expressed in ppm (δ) relative to TMS as the internal reference. *J* values are given by Hz.

(*–*)-Isopulegol **1** is commercially available from Merck Co with *ee* = 95%. (+)-Neoisopulegol **2** and (*–*)-6-methyl-3-methylenetetrahydrofuran **3** were prepared according to literature procedures. All spectroscopic data of the synthesized compounds were similar to those described therein [55]. ^1^H, ^13^C, HSQC, HMBC and NOESY NMR spectra of new compounds are available in Supplementary Materials. 

### 3.2. (2′R,3aR,6R,7aS)-6-Methylhexahydro-2H-spiro[benzofuran-3,2′-oxirane] (**5**)

*m*-chloroperbenzoic acid (70% purity, 5.87 g, 23.8 mmol) was added at 0 °C to a solution of 3 (11.9 mmol) in CH_2_Cl_2_ (50 mL) and Na_2_HPO_4_·12H_2_O (6.35 g, 35.7 mmol) in water (130 mL), and the mixture was stirred at room temperature. When the reaction was complete, as indicated by TLC (Thin layer chromatography) (2 h), the mixture was separated and the aqueous phase was extracted with CH_2_Cl_2_ (100 mL). The organic layer was washed with a 5% KOH solution (3 × 50 mL), then dried (Na_2_SO_4_) and evaporated to provide 5 as the single product.

Yield: 23%, colorless oil. [α${]}_{D}^{20}$ = −26.0 (c 0.27, MeOH). ^1^H NMR (500 MHz, CDCl_3_): δ = 0.83–0.93 (1H, m), 0.90 (3H, d, *J* = 6.5 Hz), 1.18–1.26 (1H, m), 1.32–1.42 (1H, m), 1.55–1.75 (4H, m), 2.03–2.10 (1H, m), 2.83 (1H, d, *J* = 4.2 Hz), 2.96 (1H, d, *J* = 4.2 Hz), 3.63 (1H, d, *J* = 10.6 Hz), 4.21 (1H, d, *J* = 10.6 Hz), 4.25 (1H, d, *J* = 2.4 Hz). ^13^C NMR (125 MHz, CDCl_3_): δ = 22.4, 24.7, 26.4, 33.0, 36.5, 42.7, 47.4, 68.0, 70.1, 77.9. Anal. Calculated for C_10_H_16_O_2_: C, 71.39; H, 9.59. Found: C, 71.43; H, 9.52.

### 3.3. General Procedure for Ring-Opening of Epoxide with Primary Amines

A solution of the appropriate amine (5.88 mmol) in MeCN (10 mL) and LiClO_4_ (0.31 g, 2.94 mmol) was added to a solution of epoxide **5** (0.50 g, 2.94 mmol) in MeCN (30 mL). The mixture was kept at reflux temperature for 6 h. When the reaction was completed (indicated by TLC), the mixture was evaporated to dryness, and the residue was dissolved in water (15 mL) then extracted with CH_2_Cl_2_ (3 × 50 mL). The combined organic phase was dried (Na_2_SO_4_), filtered, and concentrated. The crude product was purified by column chromatography on silica gel with an appropriate solvent mixture (CHCl_3_:MeOH = 19:1). Further purification by recrystallization from a mixture of *n*-hexane:Et_2_O resulted in compounds **7**–**10**.

#### 3.3.1. (3*R*,3a*R*,6*R*,7a*S*)-6-Methyl-3-((((*R*)-1-phenylethyl)amino)methyl)octahydrobenzofuran-3-ol (**7**)

Yield: 65%, white crystals, m.p.: 77–81 °C. [α${]}_{D}^{20}$ = +27.0 (c 0.25, MeOH). ^1^H NMR (500 MHz, CDCl_3_): δ = 0.78–0.87 (1H, m), 0.86 (3H, d, *J* = 6.5 Hz), 0.98–1.06 (1H, m), 1.12–1.18 (1H, m), 1.38 (3H, d, *J* = 6.6 Hz), 1.45–1.50 (1H, m), 1.51–1.63 (3H, m), 1.66–1.72 (1H, m), 2.00–2.05 (1H, m), 2.42 (1H, d, *J* = 12.1 Hz), 2.77 (1H, d, *J* = 12.1 Hz), 3.64 (1H, d, *J* = 9.5 Hz), 3.70 (1H, d, *J* = 9.6 Hz), 3.79 (1H, q, *J* = 6.5 Hz), 4.37 (1H, q, *J* = 3.0 Hz), 7.25–7.35 (5H, m). ^13^C NMR (125 MHz, CDCl_3_): δ = 22.4, 24.4, 24.5, 26.5, 33.2, 37.0, 47.1, 49.5, 58.6, 76.3, 77.6, 82.4, 126.7, 127.4, 128.8. Anal. Calculated for C_18_H_27_NO_2_: C, 74.70; H, 9.40; N, 4.84. Found: C, 74.73; H, 9.45; N, 4.80.

#### 3.3.2. (3*R*,3a*R*,6*R*,7a*S*)-6-Methyl-3-((((*S*)-1-phenylethyl)amino)methyl)octahydrobenzofuran-3-ol (**8**)

Yield: 75%, colorless oil. [α${]}_{D}^{20}$ = −23.0 (c 0.255, MeOH). ^1^H NMR (500 MHz, CDCl_3_): δ = 0.75–0.85 (1H, m), 0.85 (3H, d, *J* = 6.3 Hz), 0.90–1.00 (1H, m), 1.10–1.16 (1H, m), 1.35–1.40 (1H, m), 1.39 (3H, d, *J* = 6.6 Hz), 1.50–1.60 (2H, m), 1.63–1.67 (1H, m), 2.01 (1H, d, *J* = 14.5 Hz), 2.46 (1H, d, *J* = 12.2 Hz), 2.65 (1H, d, *J* = 12.2 Hz), 3.73 (3H, dd, *J* = 9.5, 20.2 Hz), 4.37 (1H, s), 7.25–7.40 (5H, m). ^13^C NMR (125 MHz, CDCl_3_): δ = 22.3, 24.2, 24.3, 26.5, 33.1, 37.0, 46.9, 49.6, 58.9, 76.2, 77.6, 82.3, 126.4, 127.4, 128.8, 144.9. Anal. Calculated for C_18_H_27_NO_2_: C, 74.70; H, 9.40; N, 4.84. Found: C, 74.68; H, 9.43; N, 4.85.

#### 3.3.3. (3*R*,3a*R*,6*R*,7a*S*)-3-((Benzylamino)methyl)-6-methyloctahydrobenzofuran-3-ol (**9**)

Yield: 78%, white crystals, m.p.: 55–56 °C. [α${]}_{D}^{20}$ = −7.0 (c 0.255, MeOH). ^1^H NMR (500 MHz, CDCl_3_): δ = 0.80–0.87 (1H, m), 0.87 (3H, d, *J* = 6.5 Hz), 1.03–1.06 (1H, m), 1.11–1.17 (1H, m), 1.45–1.49 (1H, m), 1.55–1.62 (2H, m), 2.01–2.05 (1H, m), 2.58 (1H, d, *J* = 12.1 Hz), 2.70 (1H, brs), 2.86 (1H, d, *J* = 12.2 Hz), 3.70 (1H, d, *J* = 9.5 Hz), 3.79 (1H, d, *J* = 9.6 Hz), 3.80 (1H, s), 4.39 (1H, dd, *J* = 3.0, 6.0 Hz), 7.25-7.35 (5H, m). ^13^C NMR (125 MHz, CDCl_3_): δ = 22.3, 24.3, 26.4, 33.1, 36.9, 47.1, 51.1, 54.3, 76.3, 77.6, 82.4, 127.4, 128.1, 128.6, 139.7. Anal. Calculated for C_17_H_25_NO_2_: C, 74.14; H, 9.15; N, 5.09. Found: C, 74.20; H, 9.10; N, 4.05.

#### 3.3.4. (3*R*,3a*R*,6*R*,7a*S*)-3-((Isopropylamino)methyl)-6-methyloctahydrobenzofuran-3-ol (**10**)

Yield: 83%, white crystals, m.p.: 171–173 °C. [α${]}_{D}^{20}$ = −7.0 (c 0.28, MeOH). ^1^H NMR (500 MHz, DMSO-*d*_6_): δ = 0.75–1.00 (2H, m), 0.84 (3H, d, *J* = 3.5 Hz), 1.10–1.30 (2H, m), 1.22 (6H, s), 1.48 (1H, brs), 1.57 (2H, d, *J* = 8.7 Hz), 1.75–1.95 (2H, m), 2.91 (1H, d, *J* = 12.2 Hz), 3.06 (1H, d, *J* = 12.3 Hz), 3.28 (1H, brs), 3.60 (1H, d, *J* = 8.8 Hz), 3.81 (1H, d, *J* = 8.9 Hz), 4.30 (1H, brs). ^13^C NMR (125 MHz, DMSO-*d*_6_): δ = 18.3, 18.6, 22.1, 23.2, 26.0, 32.4, 36.3, 46.1, 46.6, 50.5, 75.1., 76.4, 80.1. Anal. Calculated for C_13_H_25_NO_2_: C, 68.68; H, 11.08; N, 6.16. Found: C, 68.70; H, 11.03; N, 6.18.

### 3.4. General Procedure for Ring Closure of Aminoalcohols **7**–**10** with Formaldehyde

Thirty-five percent aqueous formaldehyde (20 mL) was added to a solution of aminoalcohols **7**–**10** (1.8 mmol) in Et_2_O (5 mL), and the mixture was stirred at room temperature. After 1 h, it was made alkaline with 10% aqueous KOH (20 mL) and extracted with Et_2_O (3 × 50 mL). After drying (Na_2_SO_4_) and solvent evaporation, crude products **12**–**15** were purified by column chromatography (CHCl_3_:MeOH = 19:1).

#### 3.4.1. (3*R*,3a*R*,6*R*,7a*S*)-6-Methyl-3′-((*R*)-1-phenylethyl)hexahydro-2H-spiro[benzofuran-3,5′-oxazolidine] (**12**)

Yield: 50%, colorless oil. [α${]}_{D}^{20}$ = +27.0 (c, 0.275 MeOH). ^1^H NMR (500 MHz, CDCl_3_): δ = 0.84–0.95 (2H, m), 0.87 (3H, d, *J* = 6.3 Hz), 1.10–1.17(1H, m), 1.34 (3H, d, *J* = 6.4 Hz), 1.50–1.65 (3H, m), 1.78–1.83 (1H, m), 2.02 (1H, d, *J* = 14.4 Hz), 2.57 (1H, d, *J* = 10.5 Hz), 2.94 (1H, d, *J* = 10.6 Hz), 3.35–3.40 (1H, m), 3.88 (2H, dd, *J* = 9.7, 19.1 Hz), 4.24–4.30 (3H, m), 7.22–7.33 (5H, m). ^13^C NMR (125 MHz, CDCl_3_): δ = 22.3, 23.4, 24.6, 26.3, 33.2, 36.9, 45.7, 53.3, 62.5, 76.4, 78.3, 84.6, 91.5, 127.2, 127.4, 128.6, 144.8. Anal. Calculated for C_19_H_27_NO_2_: C, 75.71; H, 9.03; N, 4.65. Found: C, 75.73; H, 9.00; N, 4.68.

#### 3.4.2. (3*R*,3a*R*,6*R*,7a*S*)-6-Methyl-3′-((*S*)-1-phenylethyl)hexahydro-2H-spiro[benzofuran-3,5′-oxazolidine] (**13**)

Yield: 95%, colorless oil. [α${]}_{D}^{20}$ = −27.0 (c, 0.25 MeOH). ^1^H NMR (500 MHz, CDCl_3_): δ = 0.82–0.96 (3H, m), 0.87 (3H, d, *J* = 6.3 Hz), 1.12–1.20 (1H, m), 1.25 (1H, s), 1.36 (3H, d, *J* = 6.2 Hz), 1.54–1.65 (3H, m), 1.80–1.85 (2H, m), 2.02 (1H, d, *J* = 14.4 Hz), 2.53 (1H, d, *J* = 10.6 Hz), 2.94 (1H, d, *J* = 10.7 Hz), 3.35–3.45 (1H, m), 3.87 (2H, t, *J* = 10.7 Hz), 4.26 (2H, s), 4.36 (1H, s), 7.20–7.40 (5H, m). ^13^C NMR (125 MHz, CDCl_3_): δ = 22.3, 23.5, 24.5, 26.3, 33.2, 36.8, 45.7, 53.4, 62.5, 76.2, 78.2, 84.8, 127.2, 127.4, 128.7. Anal. Calculated for C_19_H_27_NO_2_: C, 75.71; H, 9.03; N, 4.65. Found: C, 75.70; H, 9.07; N, 4.63.

#### 3.4.3. (3*R*,3a*R*,6*R*,7a*S*)-3′-Benzyl-6-methylhexahydro-2H-spiro[benzofuran-3,5′-oxazolidine] (**14**)

Yield: 90%, white crystals, m.p.: 76–77 °C. [α${]}_{D}^{20}$ = -9.0 (c, 0.25 MeOH). ^1^H NMR (500 MHz, CDCl_3_): δ = 0.84–1.00 (2H, m), 0.88 (3H, d, *J* = 6.5 Hz), 1.14–1.21 (1H, m), 1.55–1.65 (3H, m), 1.80–1.84 (1H, m), 2.04 (1H, d, *J* = 13.8 Hz), 2.70 (1H, d, *J* = 11.8 Hz), 3.09 (1H, d, *J* = 11.8 Hz), 3.68 (2H, dd, *J* = 13.0, 18.4 Hz), 3.91 (2H, dd, *J* = 9.8, 11.2 Hz), 4.31 (1H, d, *J* = 2.7 Hz), 4.35 (2H, s), 7.25–7.35 (5H, m). ^13^C NMR (125 MHz, CDCl_3_): δ = 22.3, 24.6, 26.3, 33.2, 36.8, 46.1, 54.5, 58.7, 76.7, 78.6, 86.0, 90.5, 127.5, 128.6, 128.8, 138.6. Anal. Calculated for C_18_H_25_NO_2_: C, 75.22; H, 8.77; N, 4.87. Found: C, 75.25; H, 9.73; N, 4.90.

#### 3.4.4. (3*R*,3a*R*,6*R*,7a*S*)-3′-Isopropyl-6-methylhexahydro-2H-spiro[benzofuran-3,5′-oxazolidine] (**15**)

Yield: 95%, colorless oil. [α${]}_{D}^{20}$ = −13.0 (c 0.25, MeOH). ^1^H NMR (500 MHz, DMSO-*d*_6_): δ = 0.79–0.86 (1H, m), 0.83 (3H, d, *J* = 6.6 Hz), 0.89–1.01 (2H, m), 0.99 (6H, d, *J* = 6.2 Hz), 1.07–1.13 (1H, m), 1.45–1.55 (1H, m), 1.55–1.60 (2H, m), 1.68–1.73 (1H, m), 1.86 (1H, d, *J* = 14.2 Hz), 2.35–2.40 (1H, m), 2.60 (1H, d, *J* = 10.1 Hz), 2.87 (1H, d, *J* = 10.2 Hz), 3.64 (1H, d, *J* = 9.6 Hz), 3.84 (1H, d, *J* = 9.6 Hz), 4.09 (1H, d, *J* = 2.6 Hz), 4.18 (1H, d, *J* = 3.2 Hz), 4.20 (1H, d, *J* = 3.2 Hz). ^13^C NMR (125 MHz, DMSO-*d*_6_): δ = 21.7, 21.8, 22.2, 23.7, 25.8, 32.5, 36.4, 44.9, 51.8, 51.9, 75.4, 77.1, 83.6, 91.1. Anal. Calculated for C_14_H_25_NO_2_: C, 70.25; H, 10.53; N, 5.85. Found: C, 70.28; H, 10.50; N, 5.83.

### 3.5. (3R,3aR,6R,7aS)-3-(Aminomethyl)-6-methyloctahydrobenzofuran-3-ol (**11**)

Aminoalcohols **7**–**9** (14.0 mmol) in MeOH (100 mL) were added to a suspension of palladium-on-carbon (5% Pd, 0.22 g) in MeOH (50 mL), and the mixture was stirred under an H_2_ atmosphere (1 atm) at room temperature. After the completion of the reaction (as monitored by TLC, 24 h), the mixture was filtered through a Celite pad, and the solution was evaporated to dryness. The crude product was recrystallized in Et_2_O, resulting in primary aminoalcohol **11**.

Yield: 73% (with **7**); 75% (with **8**); 70% (with **9**), white crystals, m.p.: 217–221 °C. [α${]}_{D}^{20}$ = +7.0 (c 0.25, MeOH). ^1^H NMR (500 MHz, DMSO-*d*_6_): δ = 0.74–0.89 (2H, m), 0.83 (3H, d, *J* = 5.7 Hz), 1.46 (1H, brs), 1.53–1.65 (2H, m), 1.75–1.83 (1H, m), 1.87 (1H, d, *J* = 13.8 Hz), 2.83 (1H, d, *J* = 12.9 Hz), 2.95 (1H, d, *J* = 12.9 Hz), 3.56 (1H, d, *J* = 9.2 Hz), 3.80 (1H, d, *J* = 9.2 Hz), 4.28 (1H, s), 5.45 (1H, s), 8.04 (3H, s). ^13^C NMR (125 MHz, DMSO-*d*_6_): δ = 22.2, 23.2, 25.9, 32.4, 36.4, 41.8, 45.7, 74.9, 76.5, 80.3. Anal. Calculated for C_10_H_19_NO_2_: C, 64.83; H, 10.34; N, 7.56. Found: C, 64.85; H, 10.32; N, 7.60.

### 3.6. (3R,3aR,6R,7aS)-3-(Hydroxymethyl)-6-methyloctahydrobenzofuran-3-ol (**6**)

An aqueous solution of NMO (12 mL, 50% aqueous solution) and a solution of OsO_4_ in *t*-BuOH (6 mL, 2% *t*-BuOH solution) were added in one portion to a solution of compound **3** (2.13 g, 14 mmol) in acetone (60 mL). The reaction mixture was stirred at room temperature for 24 h, then quenched by the addition of a saturated aqueous solution of Na_2_SO_3_ (100 mL), and extracted with EtOAc (Ethyl acetate, 3 × 80 mL). The organic layer was dried (Na_2_SO_4_) and evaporated. The crude product was purified by chromatography on silica gel by using *n*-hexane:EtOAc = 1:4. The product after purification was recrystallized in Et_2_O resulting in compound **6** as white crystals.

Yield: 50%, white crystals, m.p.: 67–68 °C. [α${]}_{D}^{20}$ = +3.0 (c 0.27, MeOH). ^1^H NMR (500 MHz, DMSO-*d*_6_): δ = 0.75–0.80 (1H, m), 0.82 (3H, d, *J* = 6.5 Hz), 0.94–1.03 (1H, m), 1.04–1.11 (1H, m), 1.40–1.50 (1H, m), 1.50–1.57 (2H, m), 1.62–1.67 (1H, m), 1.85 (1H, d, *J* = 14.3 Hz), 3.34–3.38 (1H, m), 3.42 (1H, d, *J* = 9.2 Hz), 3.47 (1H, dd, *J* = 5.5, 11.1Hz), 3.68 (1H, d, *J* = 9.2 Hz), 4.23 (1H, d, *J* = 2.2 Hz), 4.49 (1H, s), 4.52 (1H, t, *J* = 5.4 Hz). ^13^C NMR (125 MHz, DMSO-*d*_6_): δ = 22.3, 23.4, 26.2, 32.8, 36.7, 46.2, 63.2, 74.8, 76.5, 83.5. Anal. Calculated for C_10_H_18_O_3_: C, 64.49; H, 9.74. Found: C, 64.55; H, 9.69.

### 3.7. General Procedure for the Reaction of Benzaldehyde with Diethylzinc in the Presence of Chiral Catalysts

To the respective catalyst (0.1 mmol), 1 M Et_2_Zn in an *n*-hexane solution (3 mL, 3 mmol) was added under argon atmosphere at room temperature. The solution was stirred for 25 min at room temperature, and then benzaldehyde (1 mmol) was added. After stirring at room temperature for a further 20 h, the reaction was quenched with a saturated NH_4_Cl solution (15 mL), and the mixture was extracted with EtOAc (2 × 20 mL). The combined organic phase was washed with H_2_O (10 mL), dried (Na_2_SO_4_) and evaporated under vacuum. The obtained crude secondary alcohols were purified by flash column chromatography (*n*-hexane:EtOAc = 4:1). The *ee* and absolute configuration of the resulting material were determined by chiral GC on a Chirasil-DEX CB column after *O*-acetylation in Ac_2_O/DMPA/pyridine.

### 3.8. Antimicrobial Analyses

For the antimicrobial analyses, the pure synthesized compounds were dissolved in MeOH and diluted with H_2_O to reach concentration levels up to 400 and 40 µg/mL with a final MeOH content of 10%. Then, these test solutions were investigated in a microdilution assay with two Gram-positive bacteria (*Bacillus subtilis* SZMC 0209 and *Staphylococcus aureus* SZMC 14611), two Gram-negative bacteria (*Escherichia coli* SZMC 6271 and *Pseudomonas aeruginosa* SZMC 23290), and two yeast strains (*Candida albicans* SZMC 1533 and *C. krusei* SZMC 1352) according to the M07-A10 CLSI guideline [76] and our previous work [57,77]. For the assay, the suspensions of the microbes were prepared from overnight cultures that were cultivated in a ferment broth (bacteria: 10 g/L peptone, 5 g/L NaCl, 5 g/L yeast extract; yeast: 20 g/L peptone, 10 g/L yeast extract, and 20 g/L glucose) at 37 °C, and their concentrations were set to 2 × 10^5^ cells/mL with sterile media. Then, 96-well plates were prepared by dispensing 100 μL of suspension containing the bacterial or yeast cells, 50 μL of sterile broth, and 50 μL of the test solutions into each well, which were then incubated for 24 h at 37 °C. The mixture of 150 μL of broth and 50 μL of 10% MeOH was used as the blank sample for background correction, while 100 μL of the microbial suspension supplemented with 50 μL of the sterile broth and 50 μL of 10% MeOH was applied as the negative control. The positive control contained ampicillin (Sigma) or nystatin (Sigma) for bacteria or fungi, respectively, at two concentration levels (100 µg/mL and 10 µg/mL). The inhibitory effects of each derivative were spectrophotometrically determined at 620 nm after incubation, and the inhibition rate was calculated as the percentage of the positive control after blank correction.

## 4. Conclusions

A new library of neoisopulegol-based chiral 1,2-aminoalcohols and a diol were developed from (+)-neoisopulegol, as derived from commercially available (–)-isopulegol. The obtained aminoalcohols and diol may serve as useful building blocks for the synthesis of new heterocyclic ring systems and biologically active compounds.

The in vitro antimicrobial studies have clearly shown that the resulting *N*-substituted aminoalcohols possess moderate antibacterial action on different bacterial strains, while the diol has a remarkable antifungal effect.

Aminoalcohol derivatives were also applied as chiral catalysts in the enantioselective addition of diethylzinc to benzaldehyde with moderate but opposite enantioselectivity.

## Figures and Tables

**Table 1:** Addition of diethylzinc to benzaldehyde catalyzed by aminoalcohol derivatives.

Entry	Ligand	Yield ^a^ (%)	*ee*^b^ (%)	Configuration of the Major Product ^c^
1	**7**	86	8	*(R)*
2	**8**	90	40	*(S)*
3	**9**	89	11	*(S)*
4	**10**	93	4	*(S)*
5	**11**	95	7	*(S)*
6	**12**	88	11	*(R)*
7	**13**	90	3	*(R)*
8	**14**	82	6	*(R)*
9	**15**	80	7	*(R)*

^a^ After silica column chromatography. ^b^ Determined using the crude product by GC analysis (Chirasil-DEX CB column). ^c^ Determined by comparing the t_R_ of GC analysis and optical rotations with literature data.

**Table 2:** Antimicrobial activities of the synthesized compounds.

					Inhibitory effect (%) ± RSD (%)		
		*Yeast*		*Gram-Negative*		*Gram-Positive*	
Analogue	Conc. (µg/mL)	*C. albicans*	*C. krusei*	*E. coli*	*P. aeruginosa*	*B. subtilis*	*S. aureus*
6	10	−	36.5 ± 8.43	−	−	−	−
	100	−	58.4 ± 14.41	−	−	21.7 ± 6.05	−
7	10	−	−	8.7 ± 3.15	7.5 ± 1.54	−	−
	100	−	−	20.0 ± 2.81	8.7 ± 0.49	−	7.1 ± 4.3
8	10	−	−	−	−	19.0 ± 2.61	−
	100	−	−	17.1 ± 4.94	5.3 ± 4.31	31.9 ± 2.74	−
9	10	−	−	16.7 ± 6.68	9.9 ± 1.8	−	−
	100	−	−	21.0 ± 5.05	31.6 ± 1.73	9.8 ± 11.2	13.8 ± 1.73
10	10	−	−	3.7 ± 1.68	−	−	−
	100	−	−	4.3 ± 10.71	2.3 ± 5.93	10.5 ± 10.12	−
11	10	−	3.7 ± 0.04	−	−	−	−
	100	−	16.0 ± 14.5	−	−	−	−
12	10	−	−	15.3 ± 4.35	−	−	9.2 ± 7.75
	100	−	−	26.2 ± 4.06	1.8 ± 6.28	−	20.2 ± 8.92
13	10	−	−	17.1 ± 8.19	−	−	−
	100	−	−	27.7 ± 8.54	7.0 ± 4.62	−	3.9 ± 3.39
14	10	−	−	14.6 ± 4.38	4.1 ± 7.10	−	12.6 ± 0.57
	100	−	−	25.3 ± 2.99	16.8 ± 5.69	−	14.0 ± 3.68
15	10	−	−	5.1 ± 7.92	−	−	−
	100	−	−	14.8 ± 4.87	−	1.5 ± 11.4	−

## Supplementary Materials

The following are available online, Figures S3–S31: 1H, 13C, HSQC, HMBC and NOESY NMR spectra of new compounds.
