# Supplementary material for: Synthesis and Application of 1,2-Aminoalcohols with Neoisopulegol-Based Octahydrobenzofuran Core
Source: Molecules. 2019 Dec 19;25(1):21. doi: 10.3390/molecules25010021 (PMC6982906; doi:10.3390/molecules25010021)

Supporting information for

Synthesis and application of 1,2-aminoalcohols with neoisopulegol-based octahydrobenzofuran core

Fatima Zahra Bamou[1], Tam Minh Le [1,2], Bettina Volford [3], András Szekeres [3] and Zsolt Szakonyi [1,4*]

[1] Institute of Pharmaceutical Chemistry, University of Szeged, Interdisciplinary excellent center, H-6720 Szeged, Eötvös utca 6, Hungary; fatima@pharm.u-szeged.hu; leminhtam@pharm.u-szeged.hu

[2] MTA-SZTE Stereochemistry Research Group, Hungarian Academy of Sciences, H-6720 Szeged, Eötvös utca 6, Hungary

[3] Department of Microbiology, University of Szeged, 6726 Szeged, Közép fasor 52, Hungary; bettina.volford86@gmail.com; andras.j.szekeres@gmail.com

[4] Interdisciplinary Centre of Natural Products, University of Szeged, H-6720 Szeged, Eötvös utca 6, Hungary

* Correspondence: szakonyi@pharm.u-szeged.hu; Tel.: +36-62-546809; Fax: +36-62-545705

Contents

^{1}H, ^{13}C, HSQC, HMBC and NOESY NMR spectra of new compounds S3 – S31

^{1}H-NMR of compound **5**

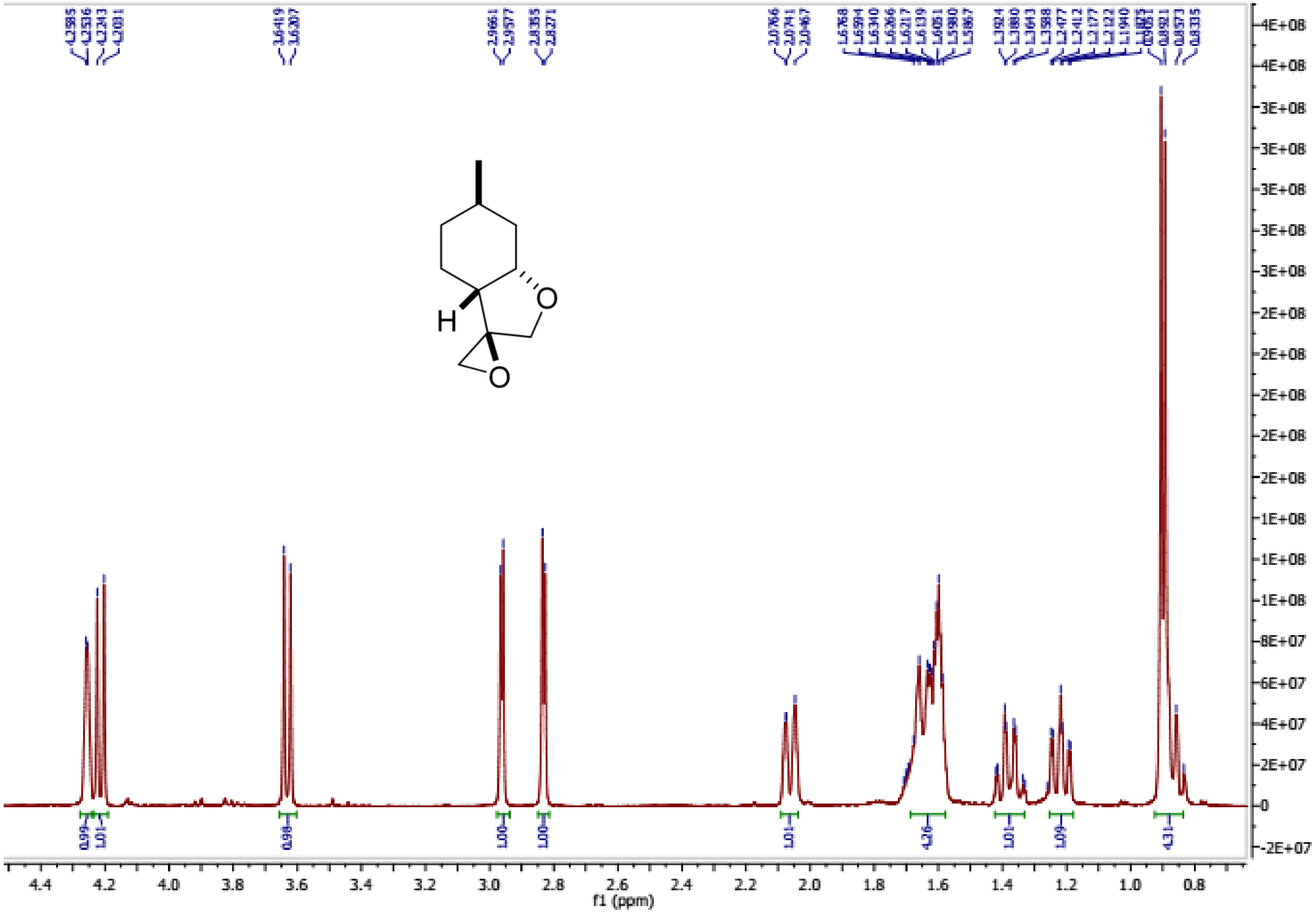

^{13}C-NMR of compound **5**

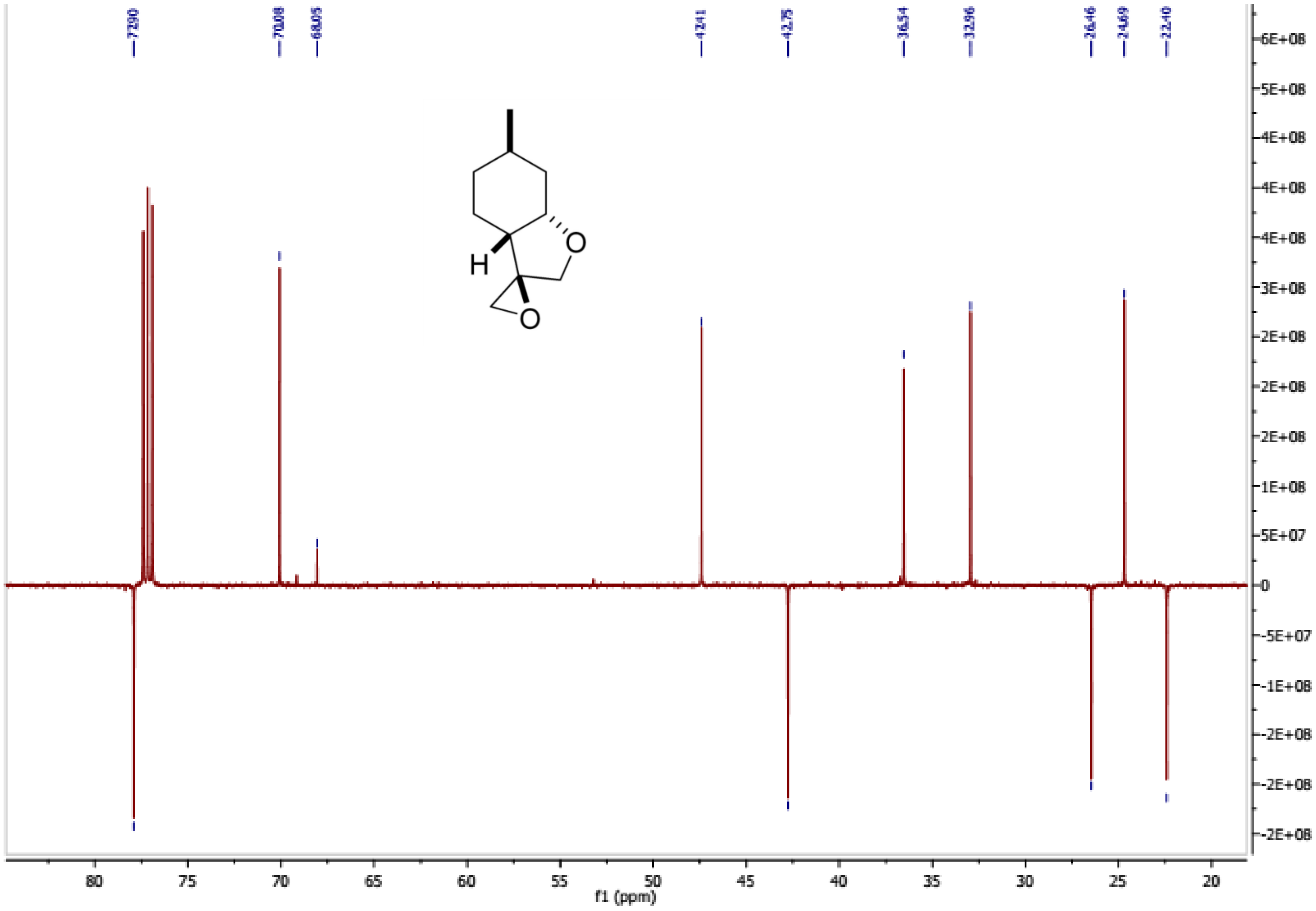

^{1}H-NMR of compound **6**

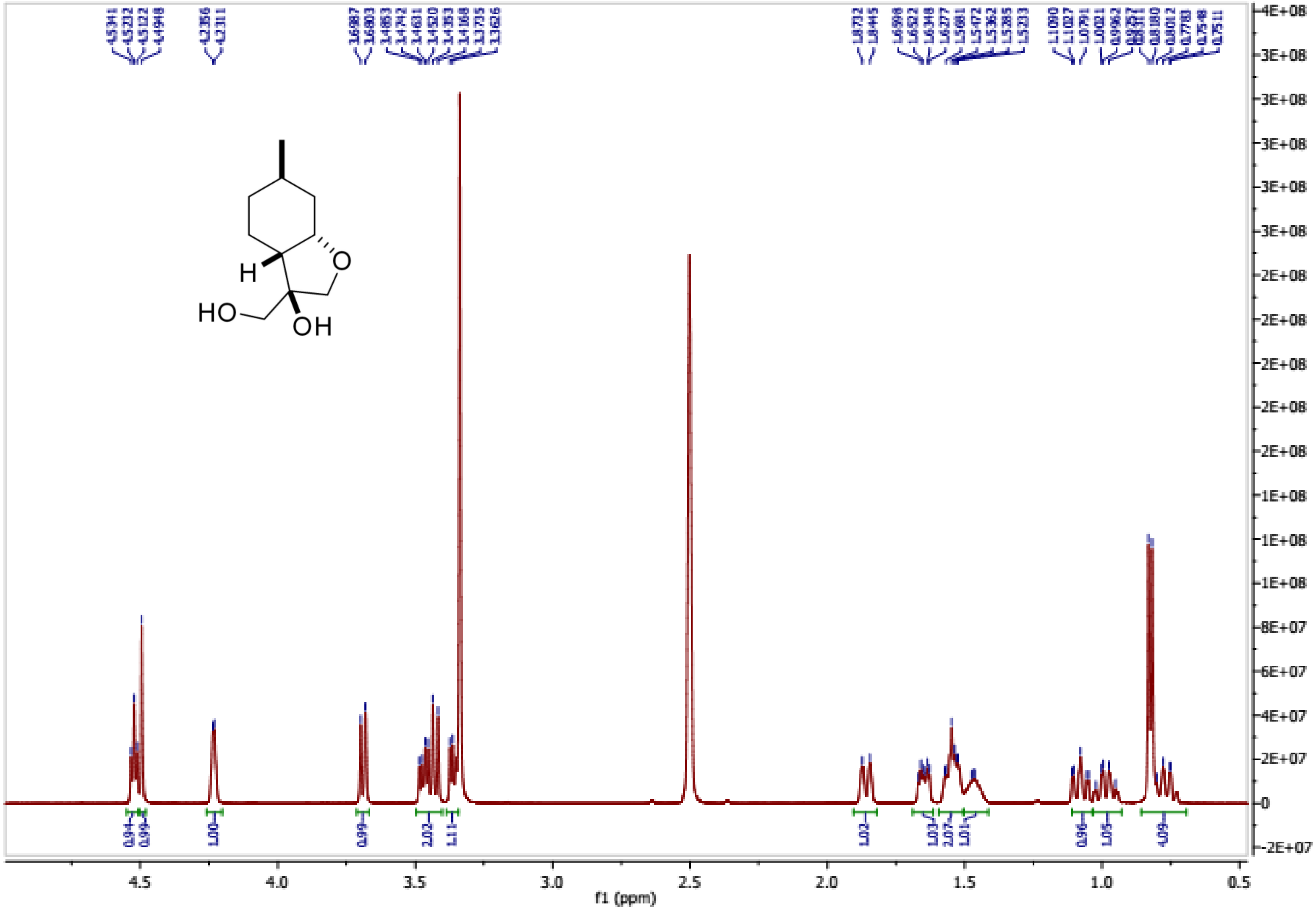

^{13}C-NMR of compound **6**

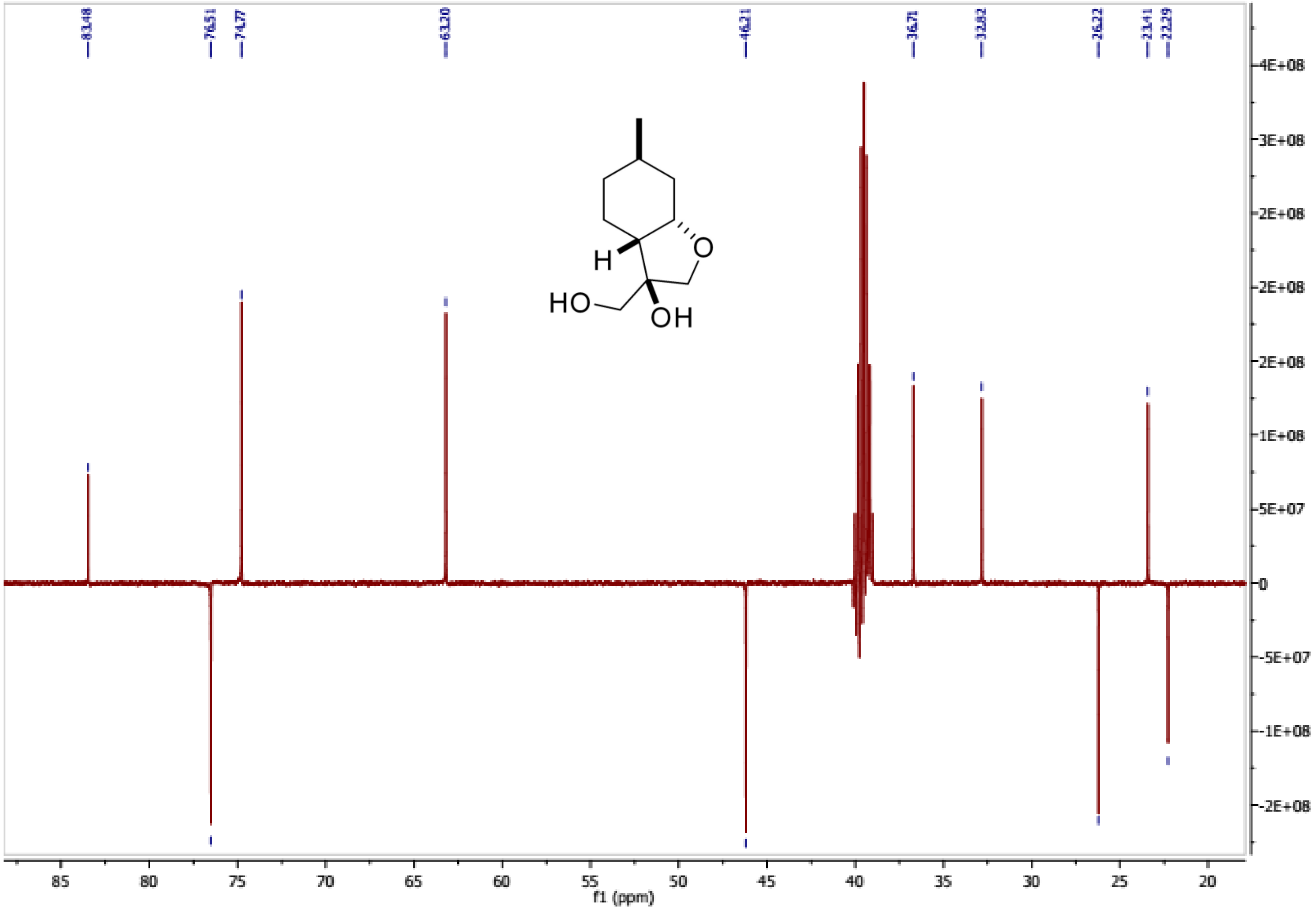

NOESY of compound **6**

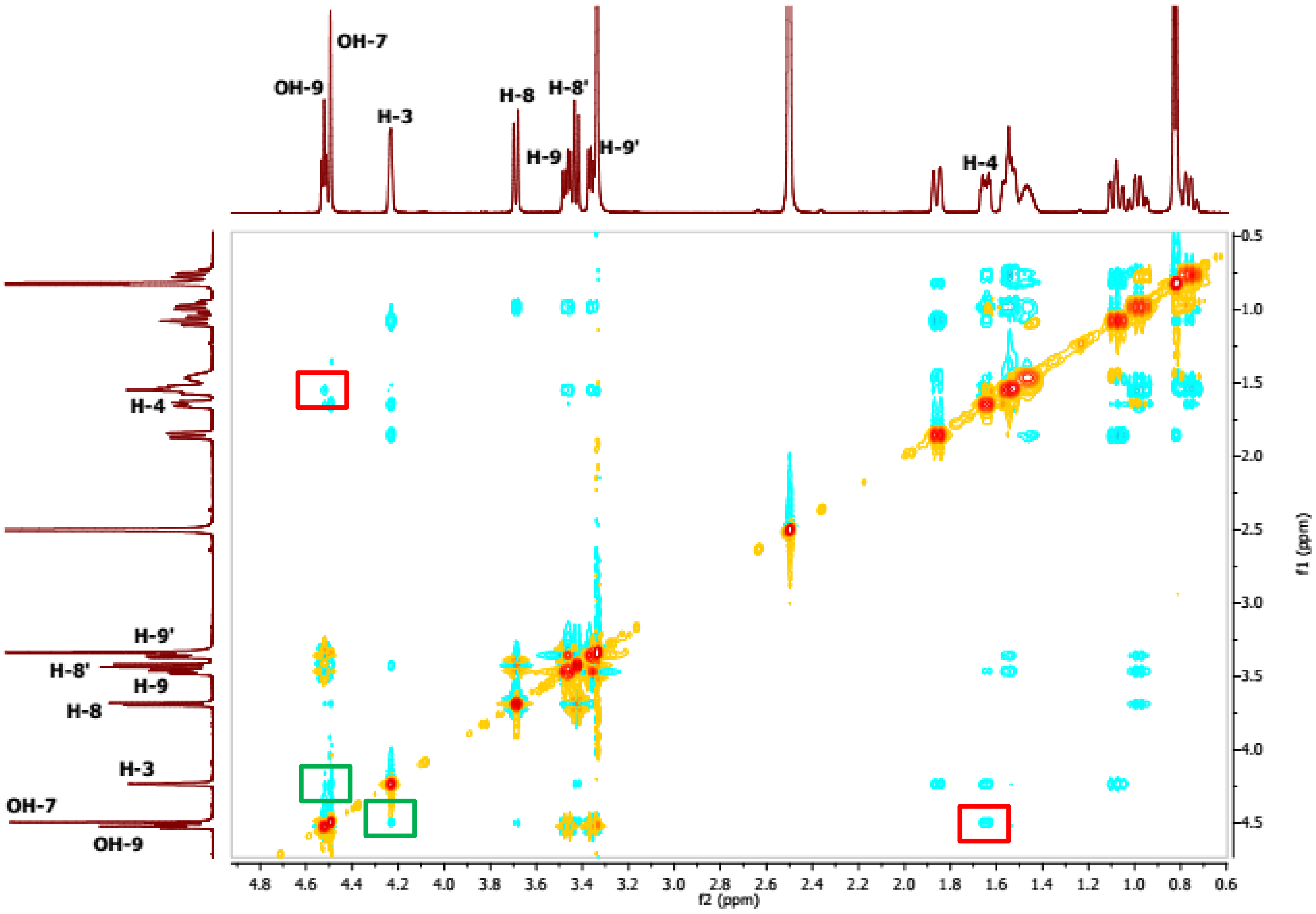

HSQC of compound **6**

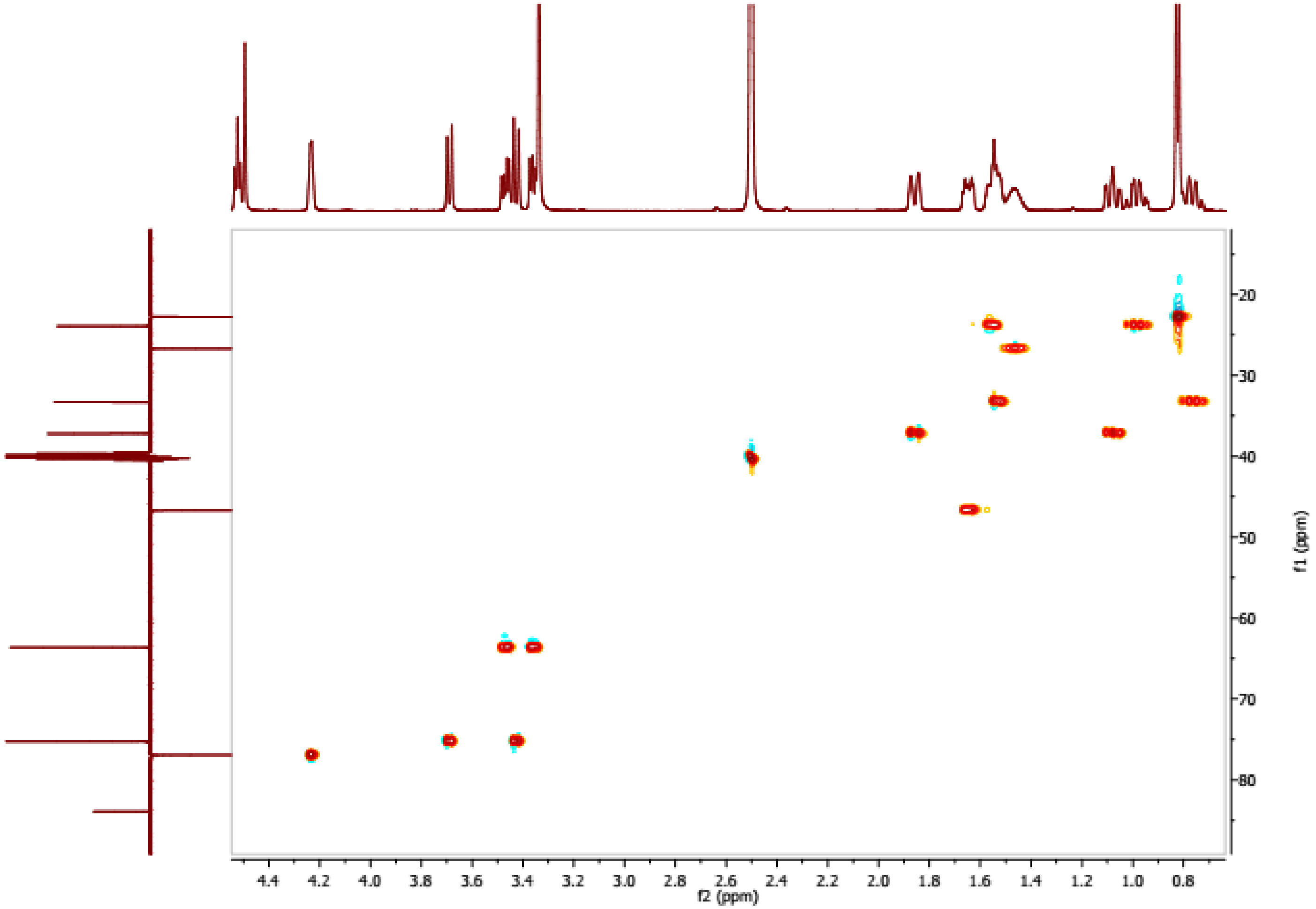

HMBC of compound **6**

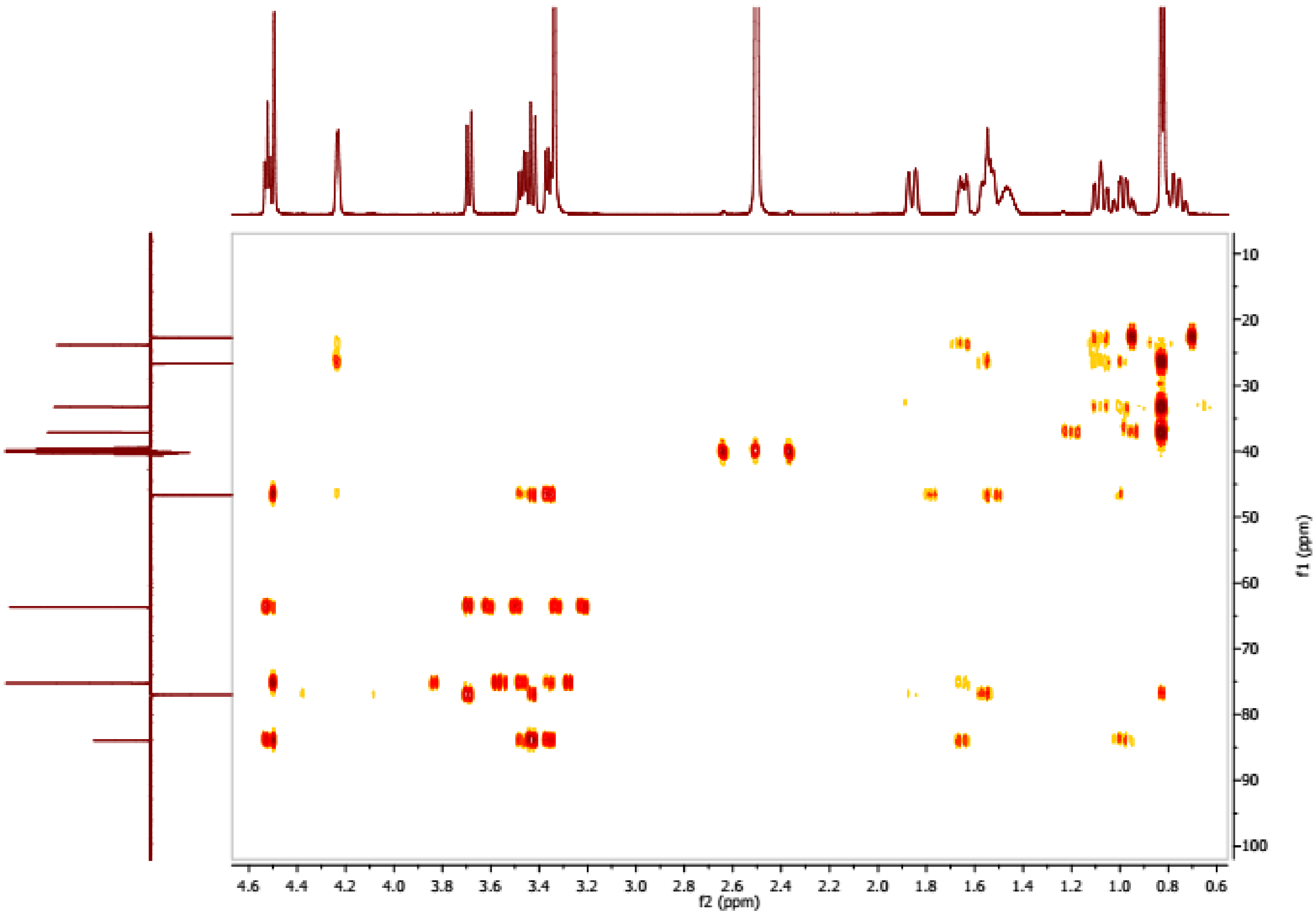

^{1}H-NMR of compound **7**

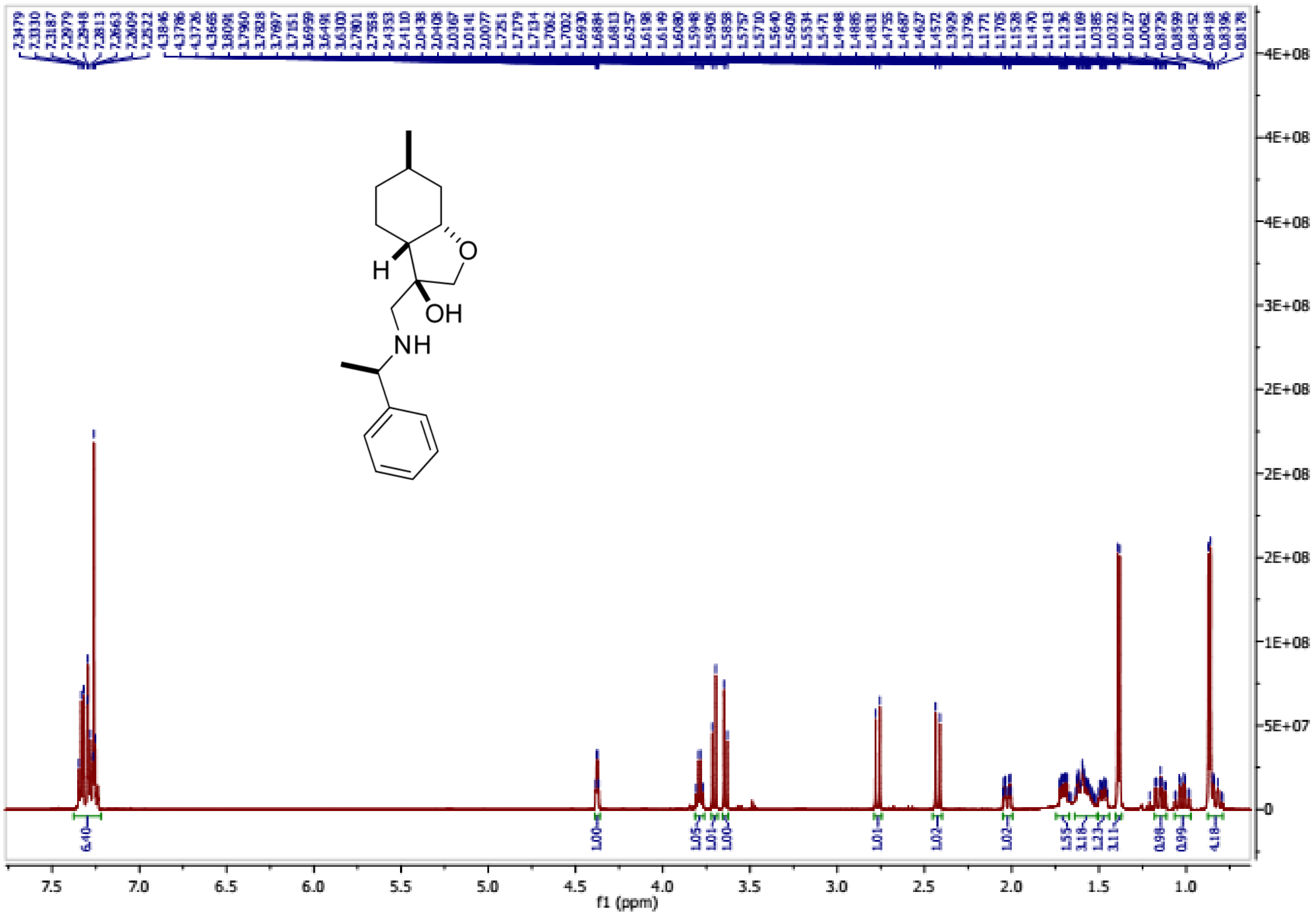

^{13}C-NMR of compound **7**

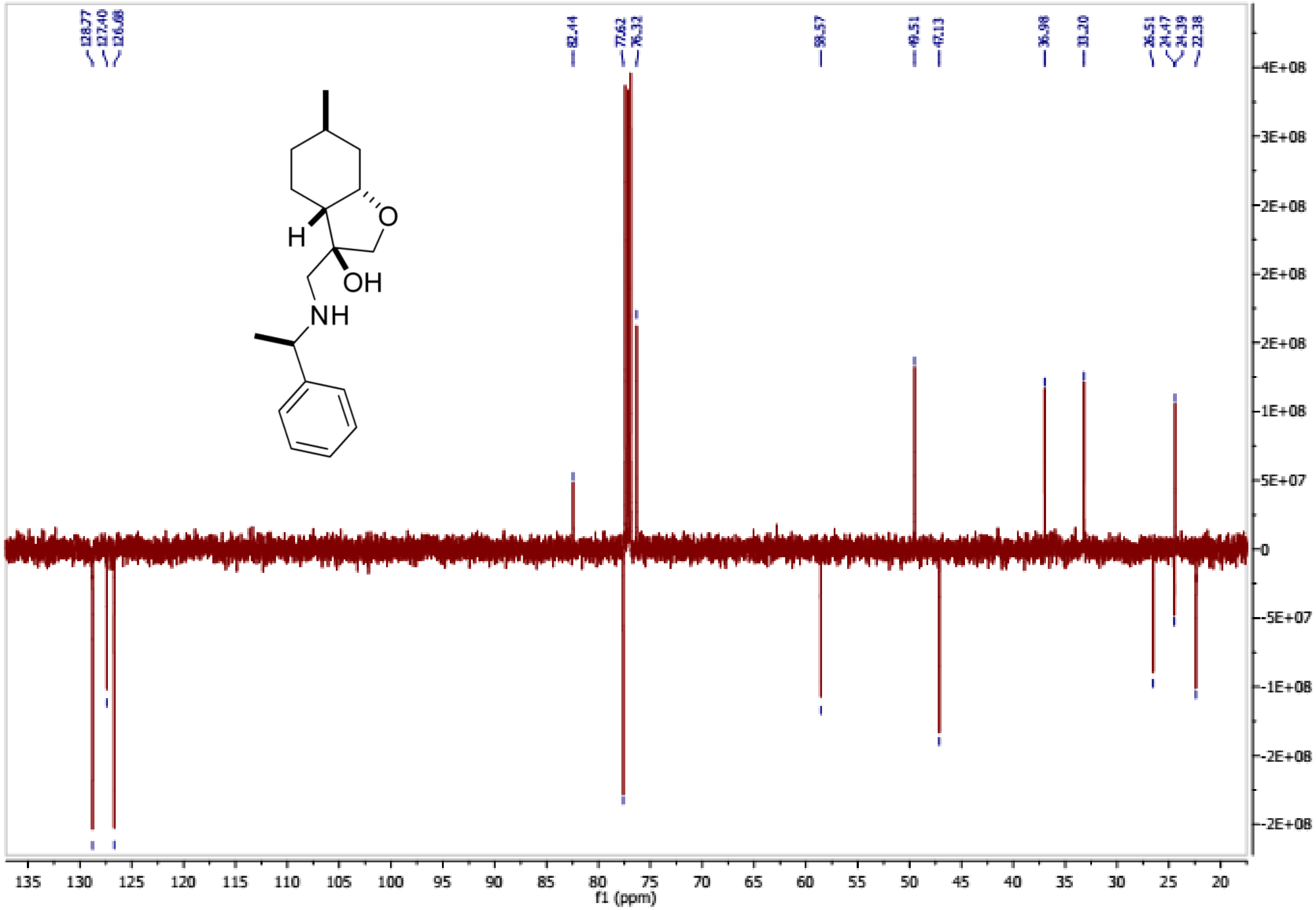

^{1}H-NMR of compound **8**

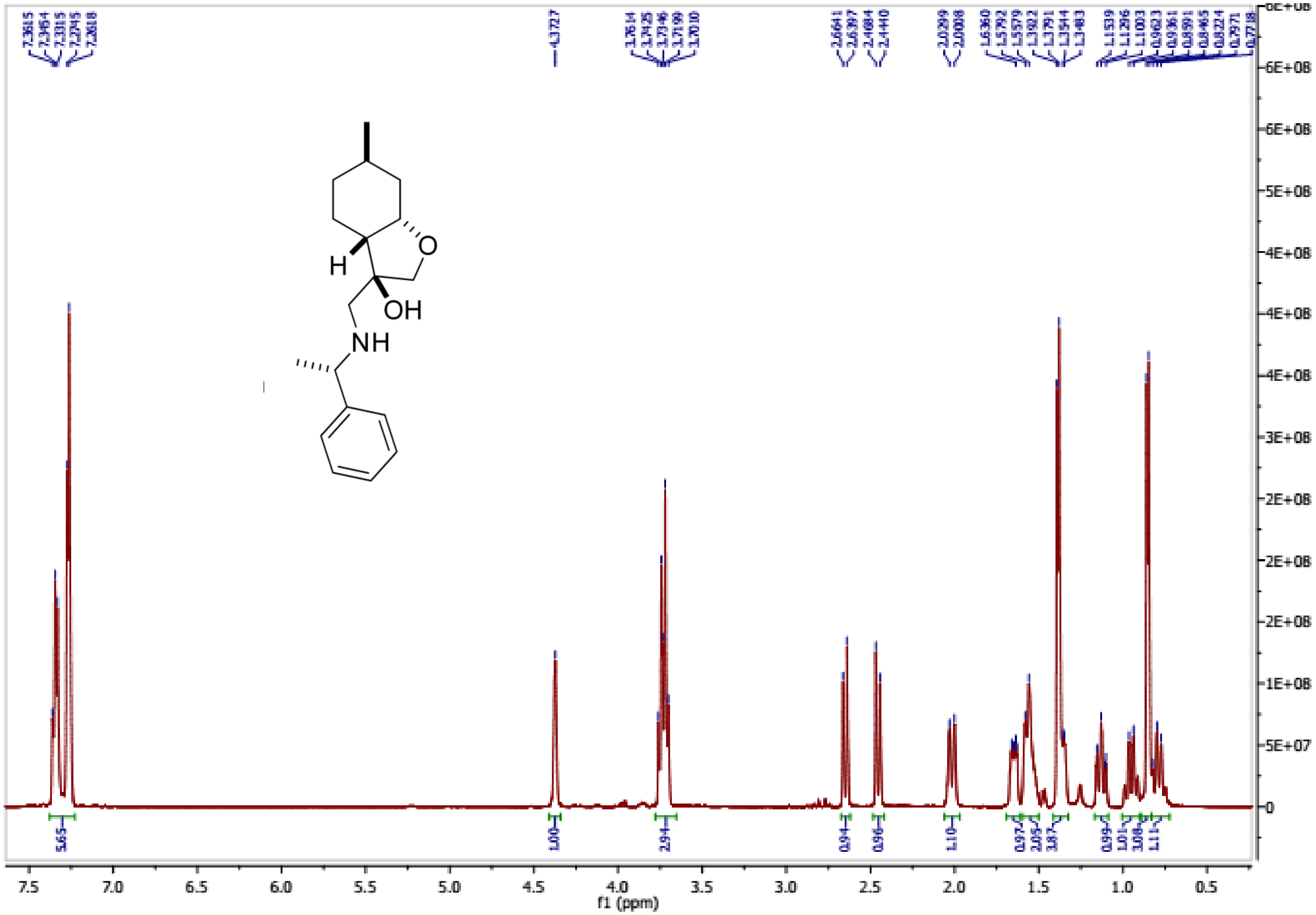

^{13}C-NMR of compound **8**

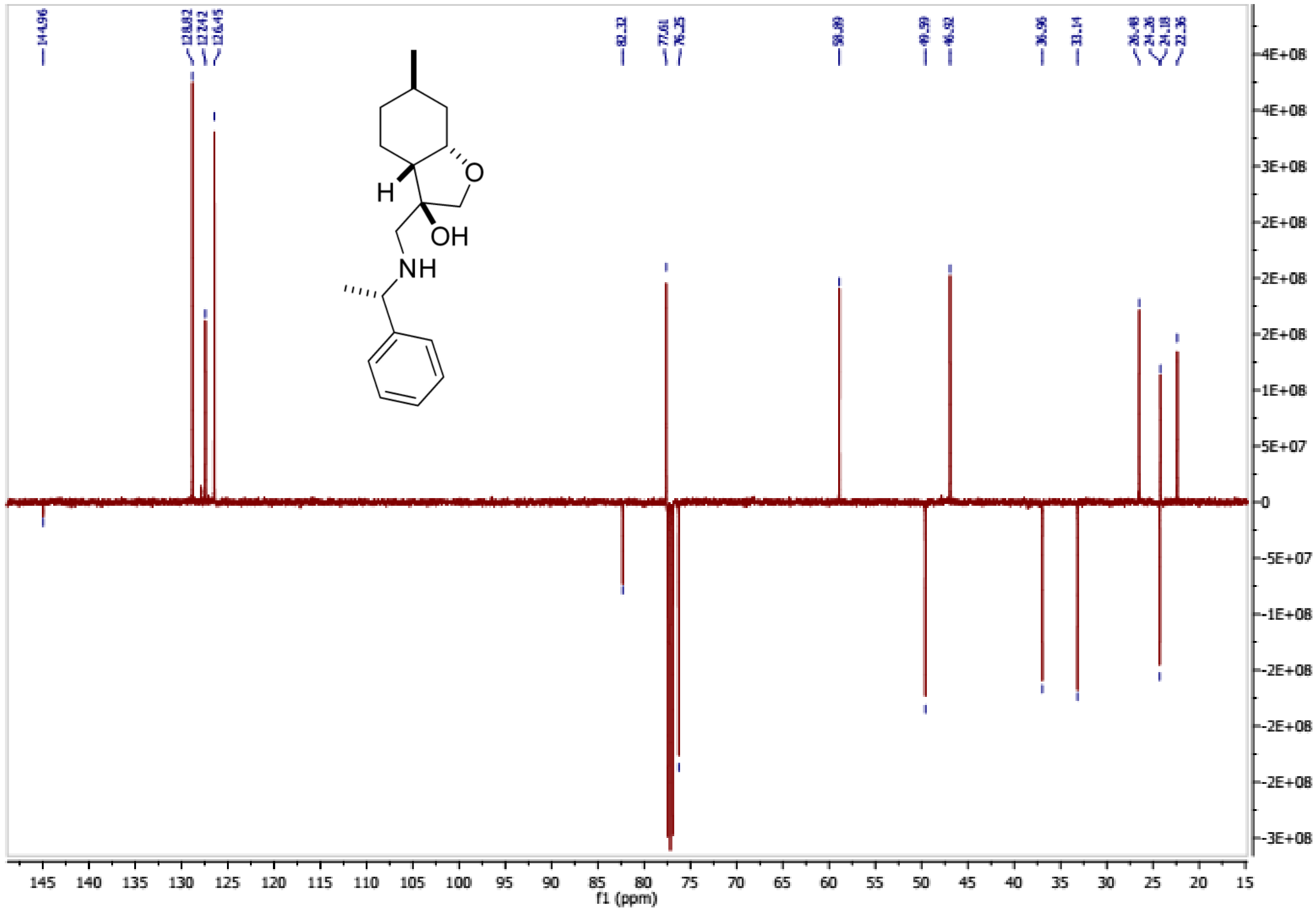

^{1}H-NMR of compound **9**

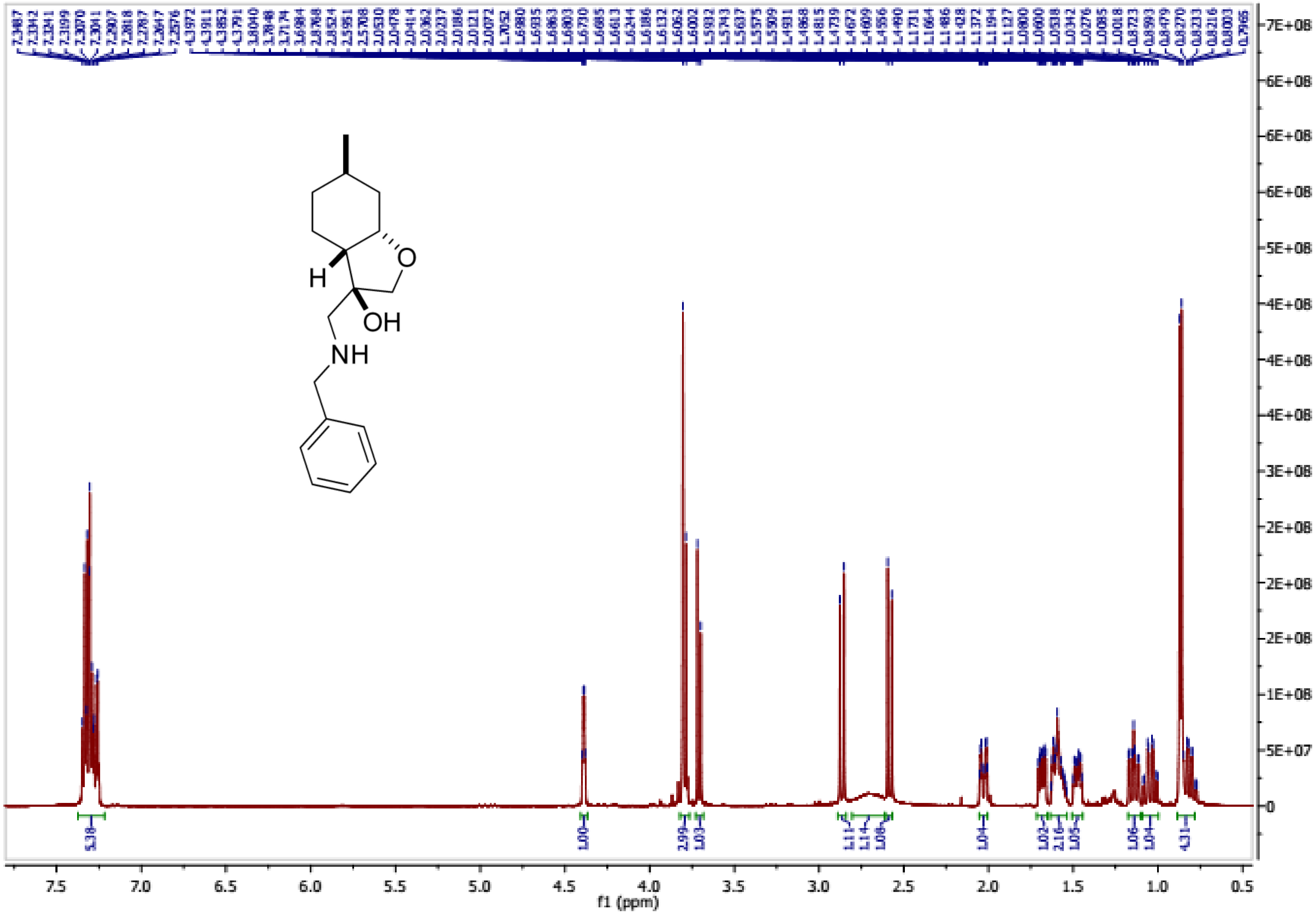

^{13}C-NMR of compound **9**

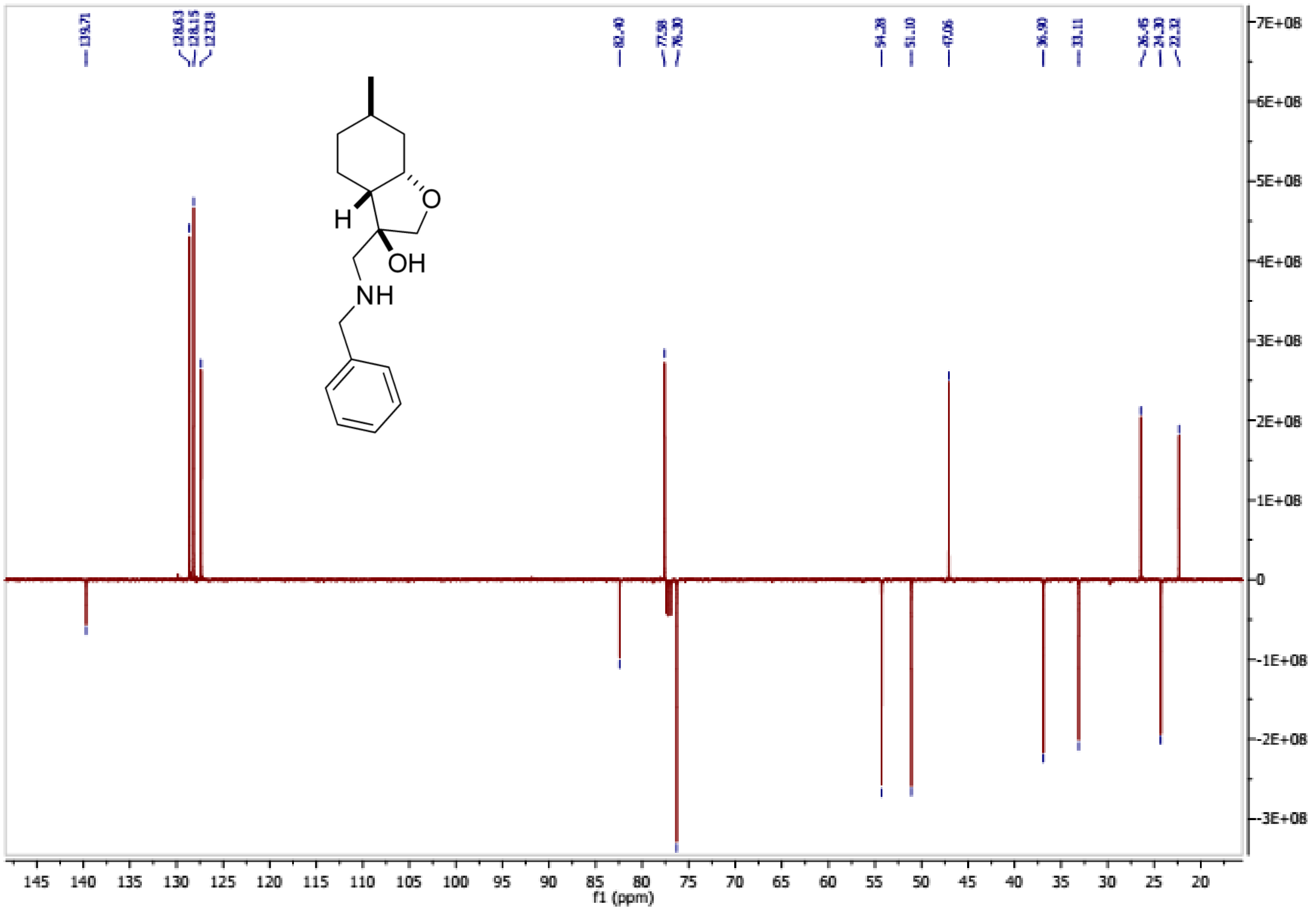

^{1}H-NMR of compound **10**

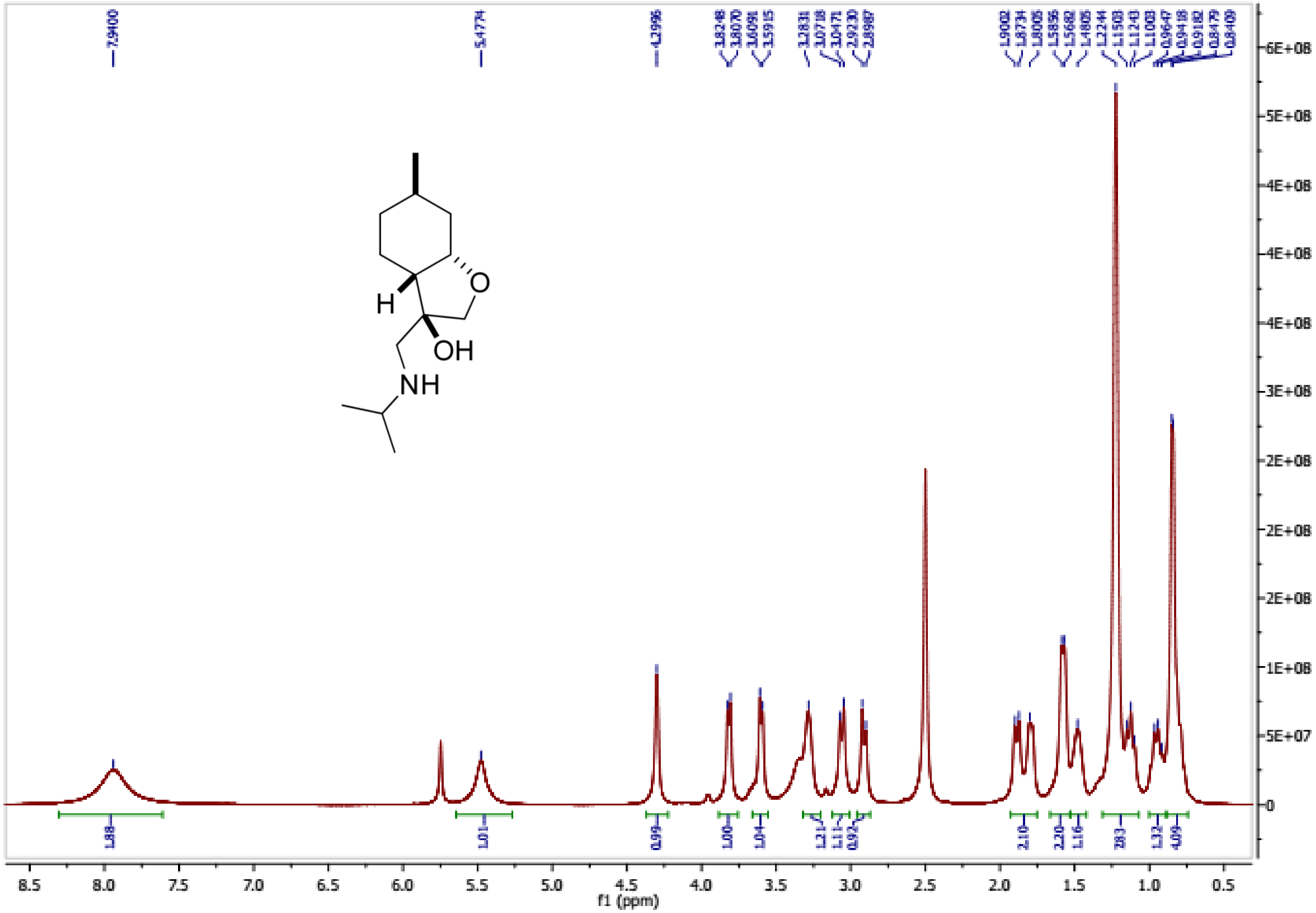

^{13}C-NMR of compound **10**

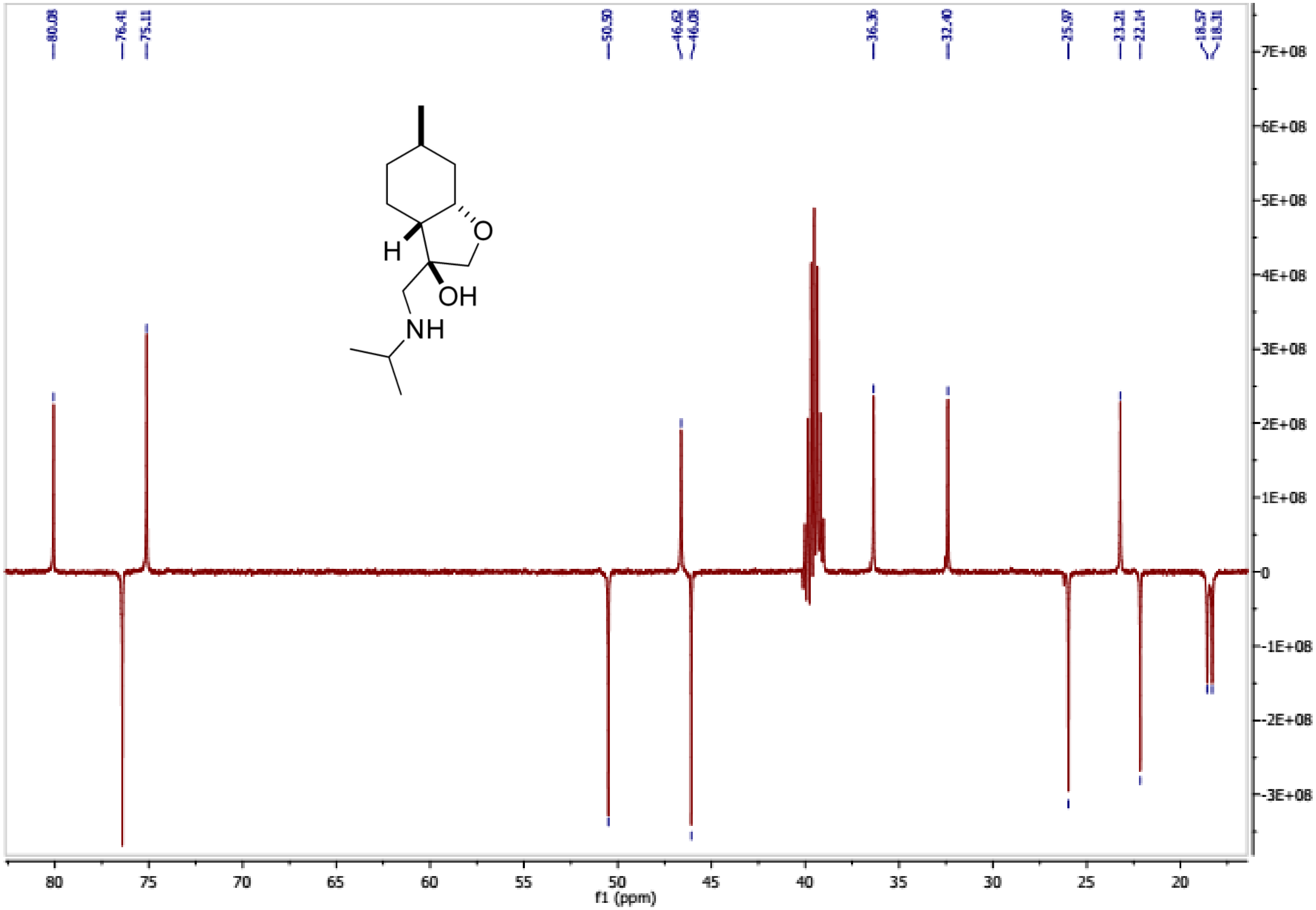

COSY of compound **10**

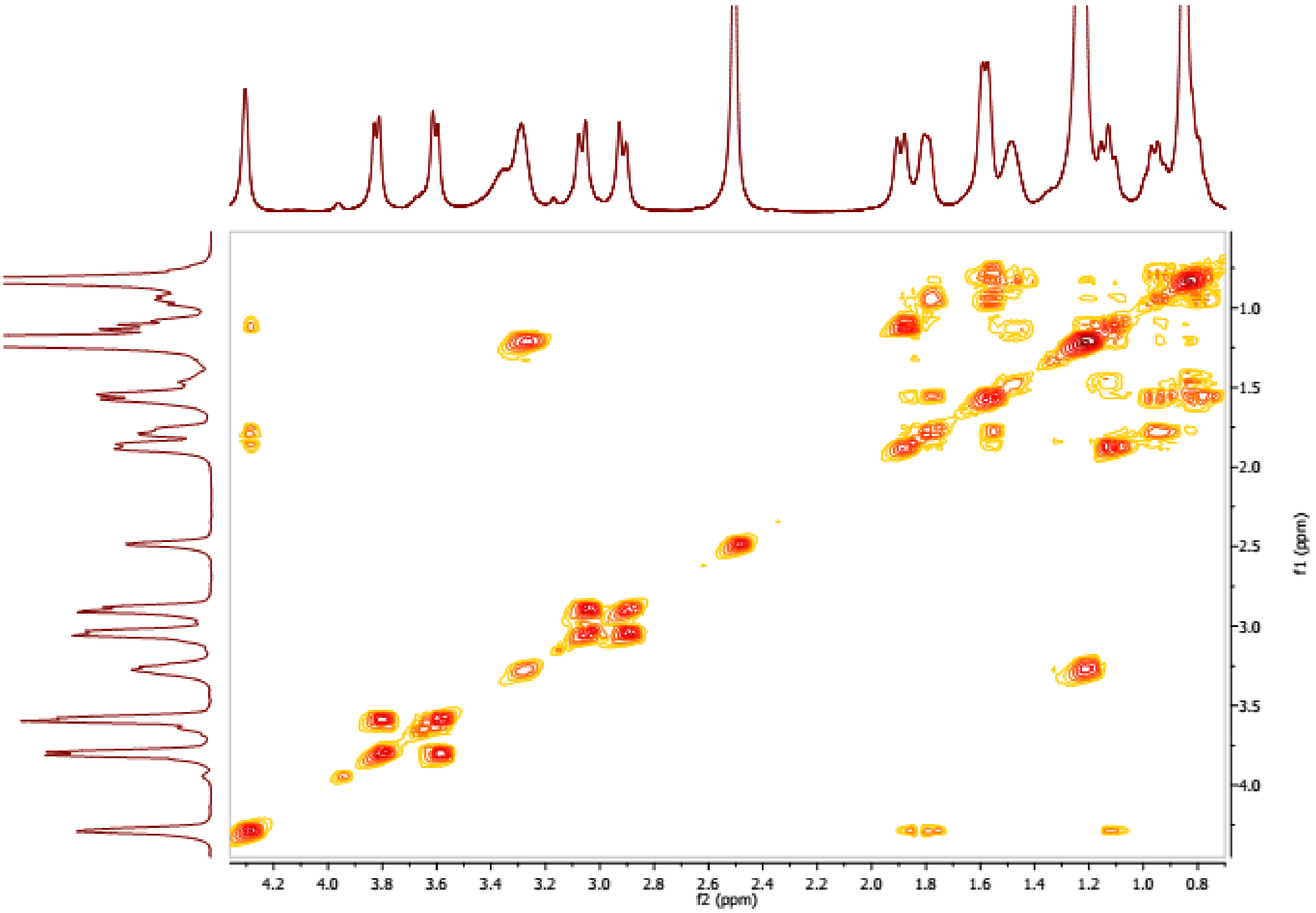

NOESY of compound **10**

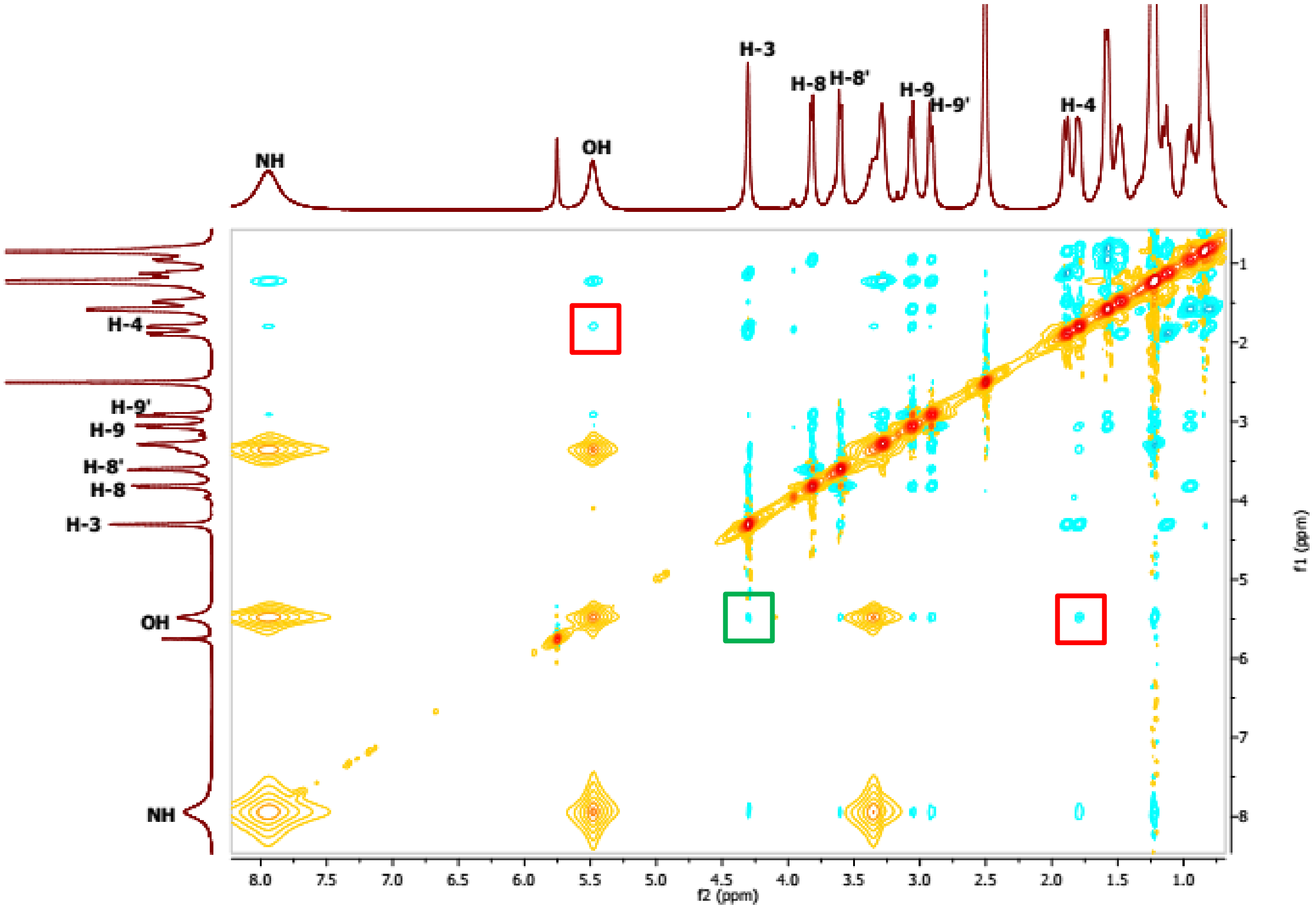

HSQC of compound **10**

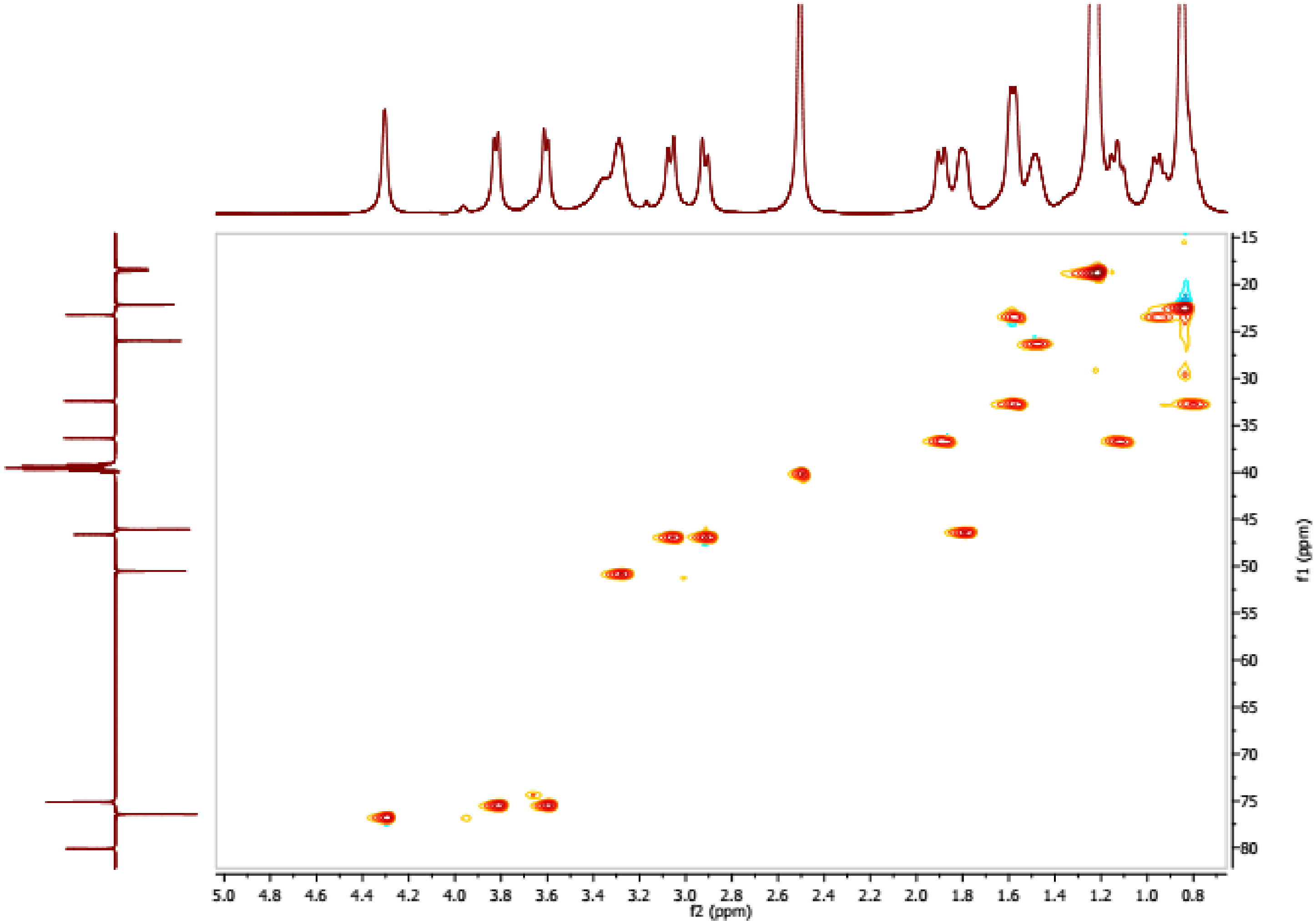

HMBC of compound **10**

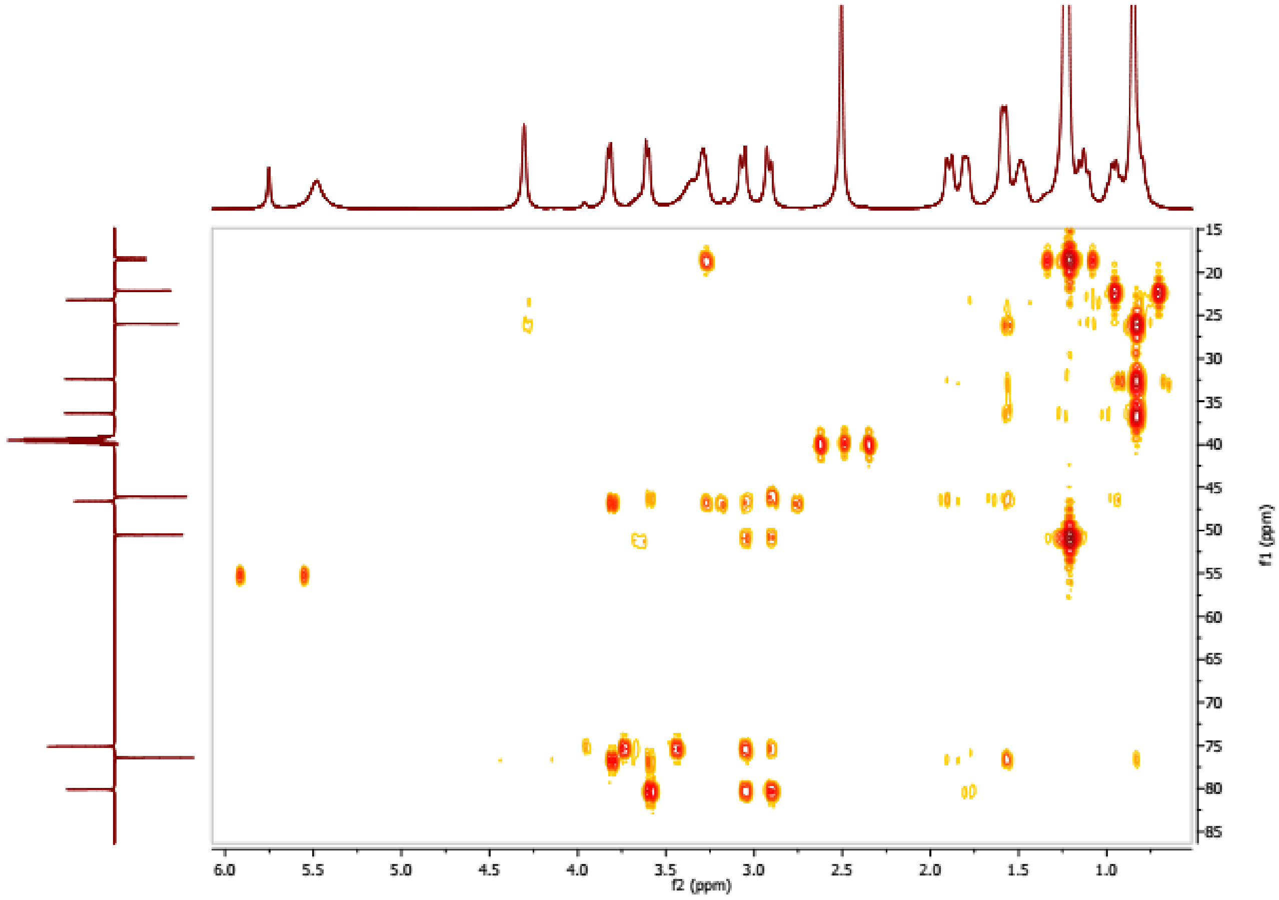

^{1}H-NMR of compound **11**

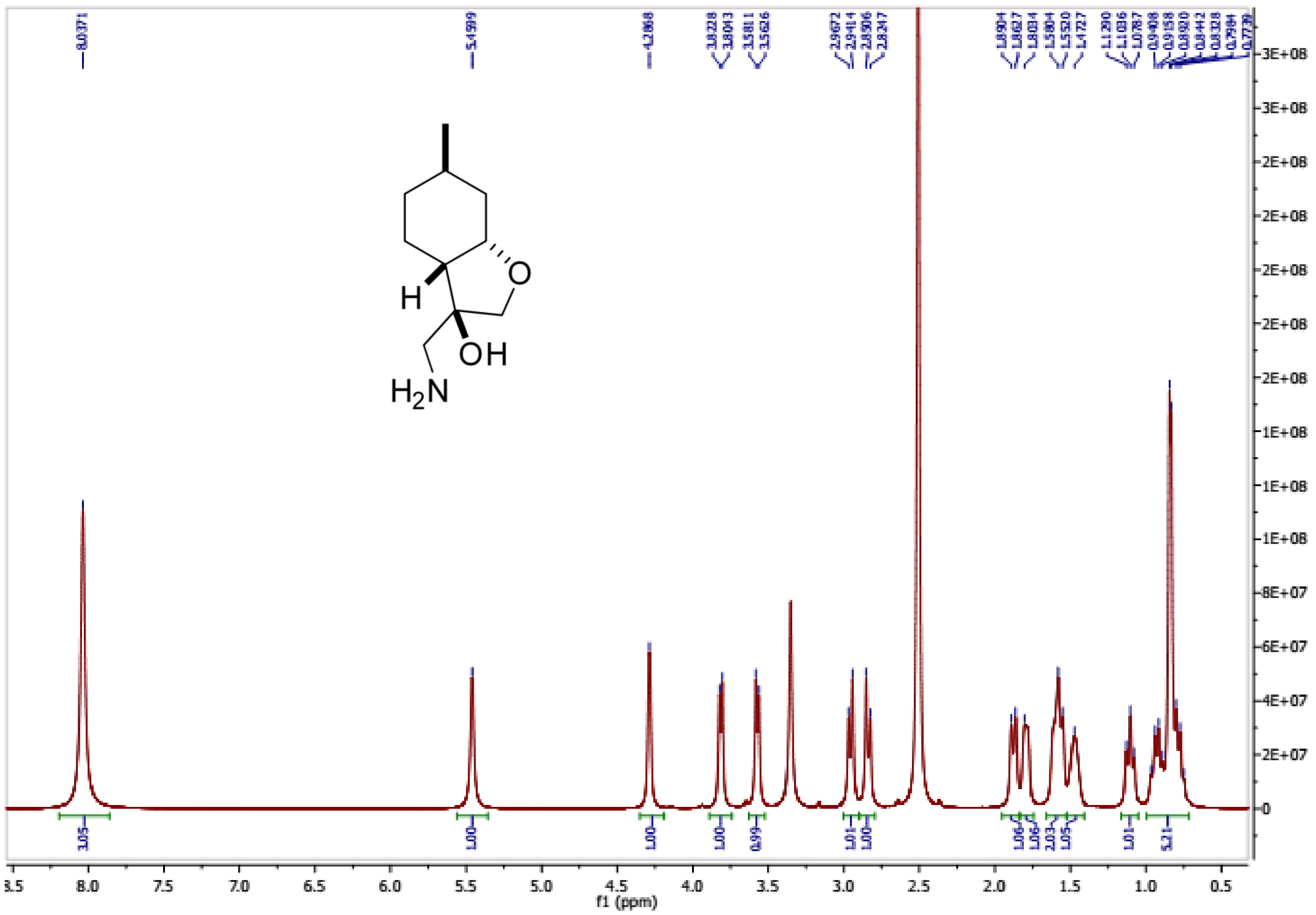

^{13}C-NMR of compound **11**

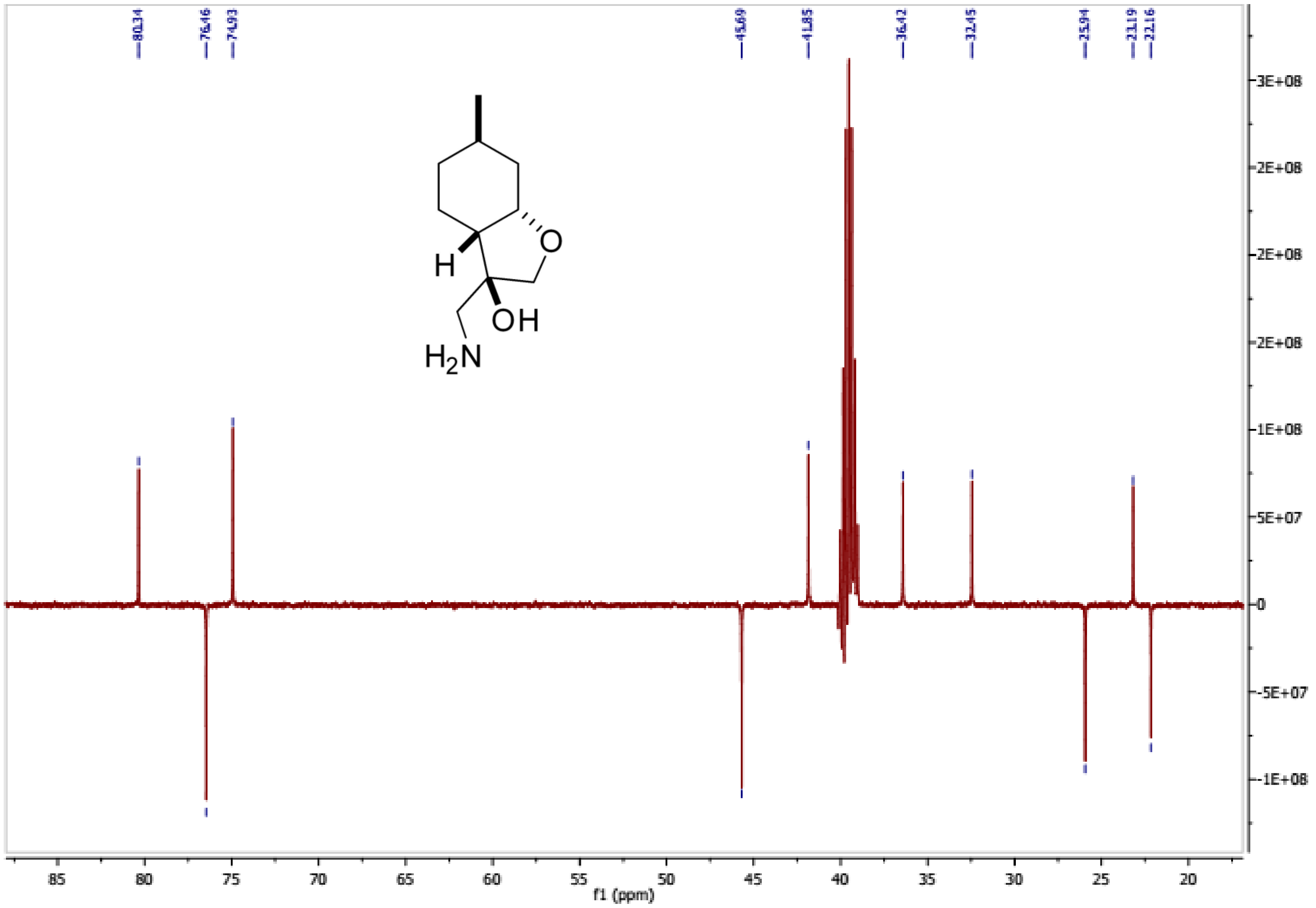

^{1}H-NMR of compound **12**

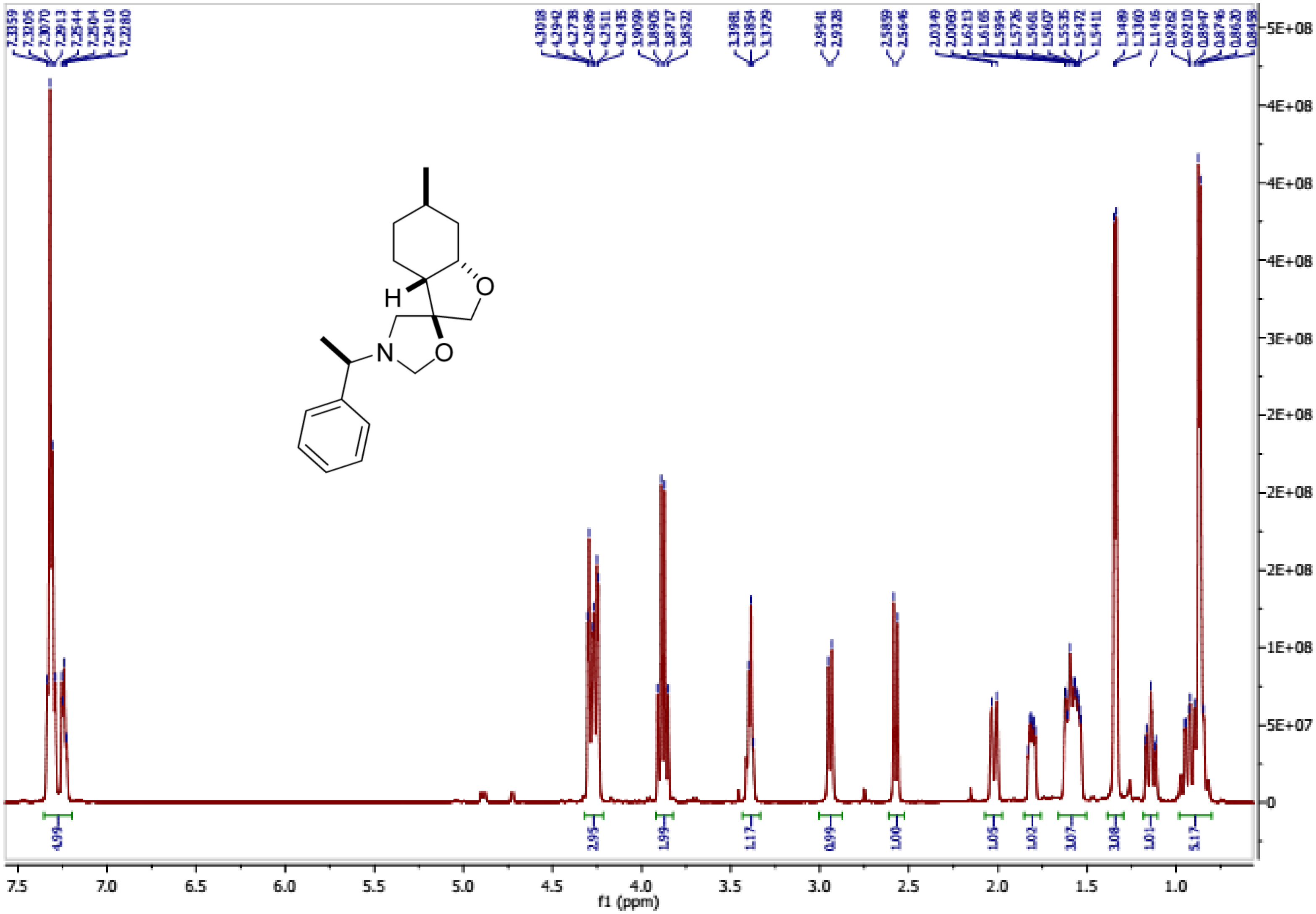

13C-NMR of compound **12**

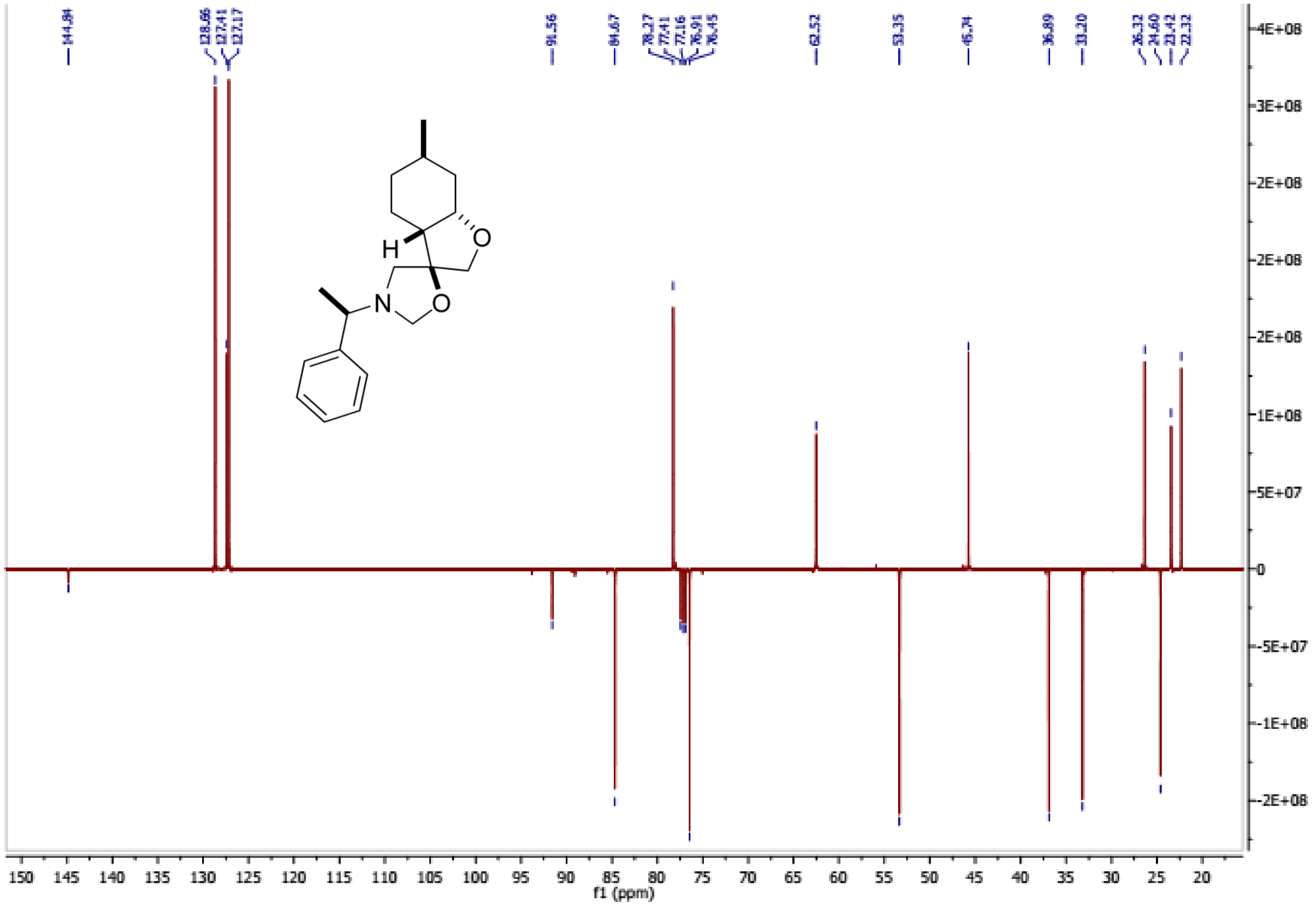

^{1}H-NMR of compound **13**

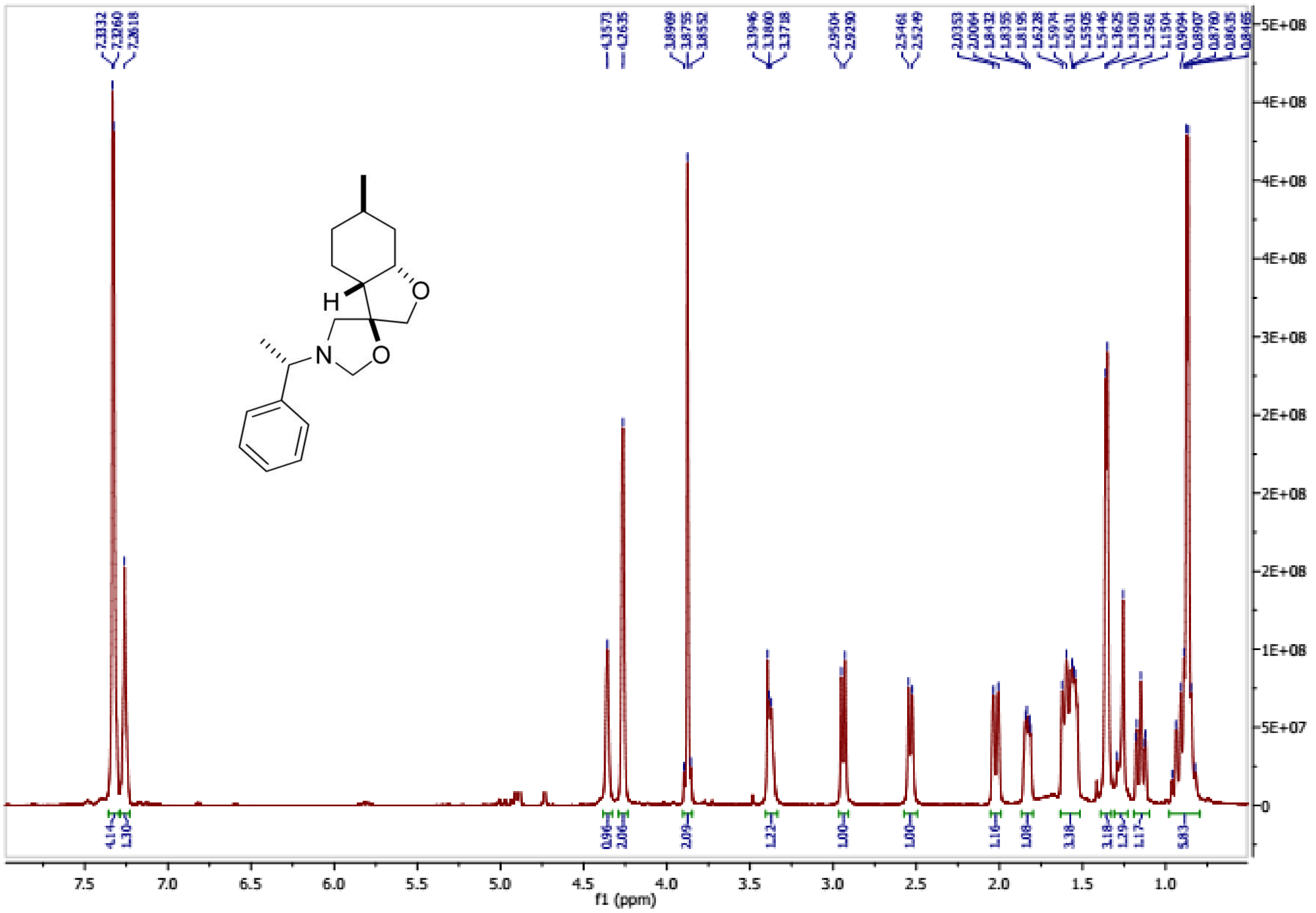

13C-NMR of compound **13**

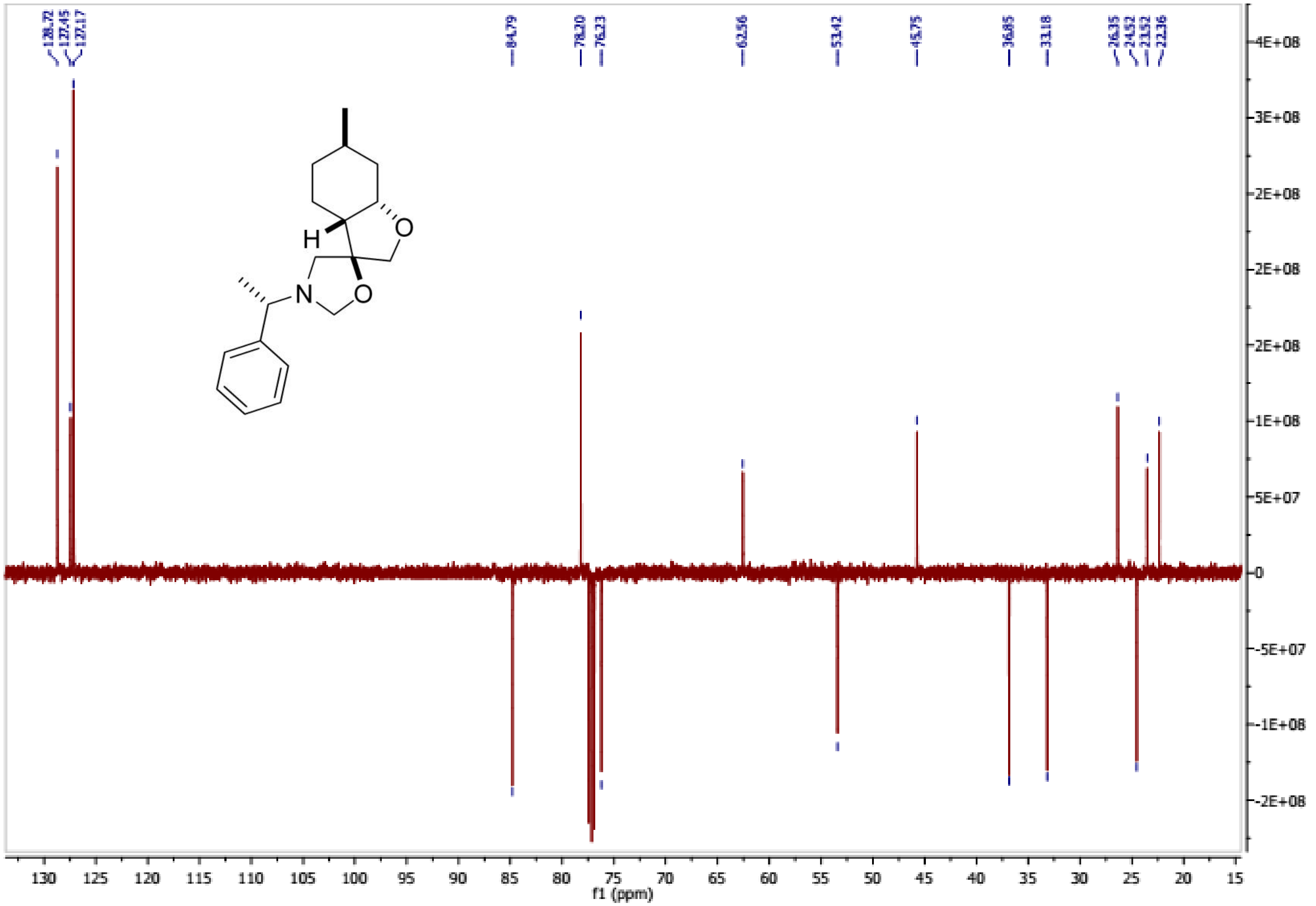

^{1}H-NMR of compound **14**

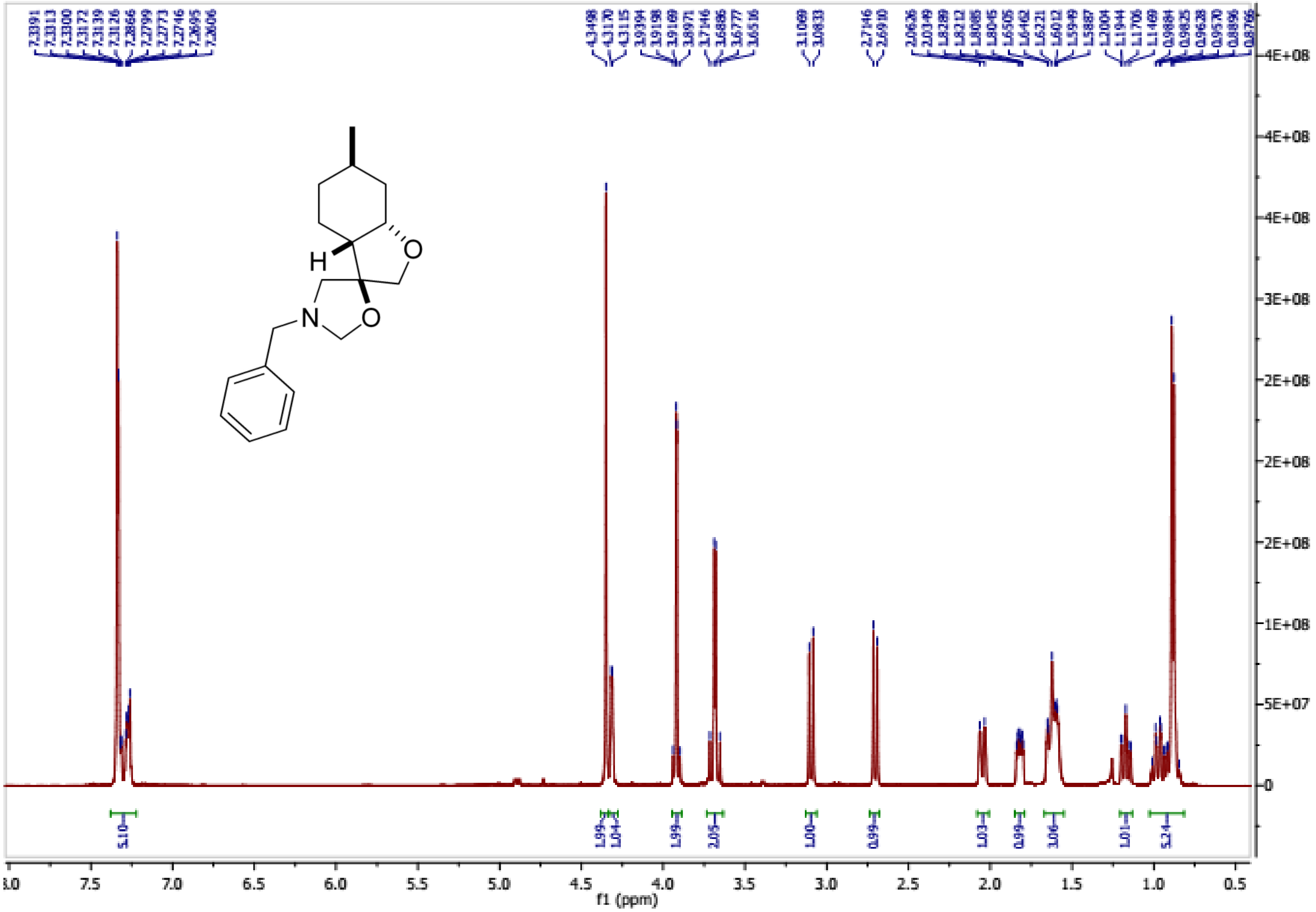

^{13}C-NMR of compound **14**

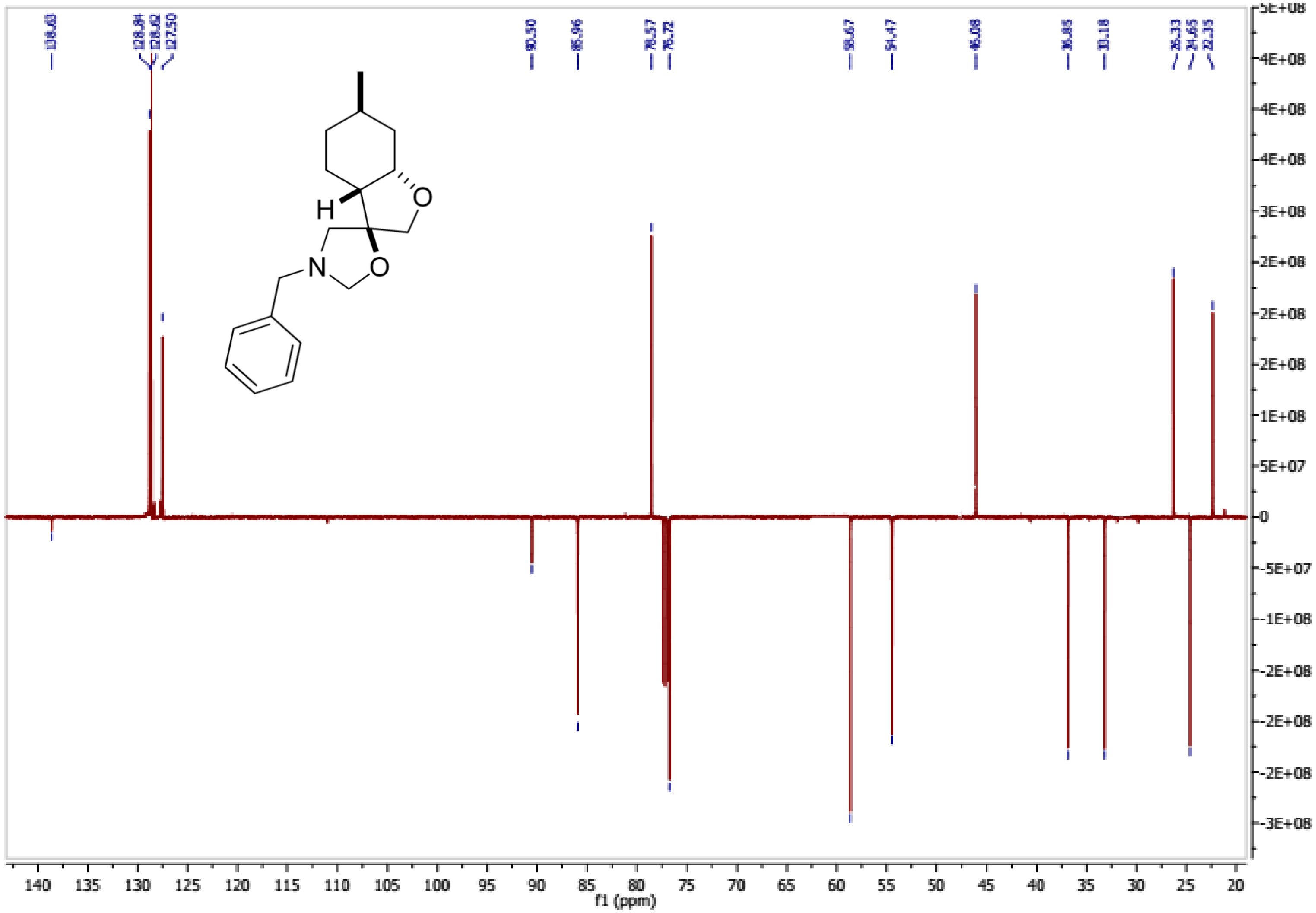

^{1}H-NMR of compound **15**

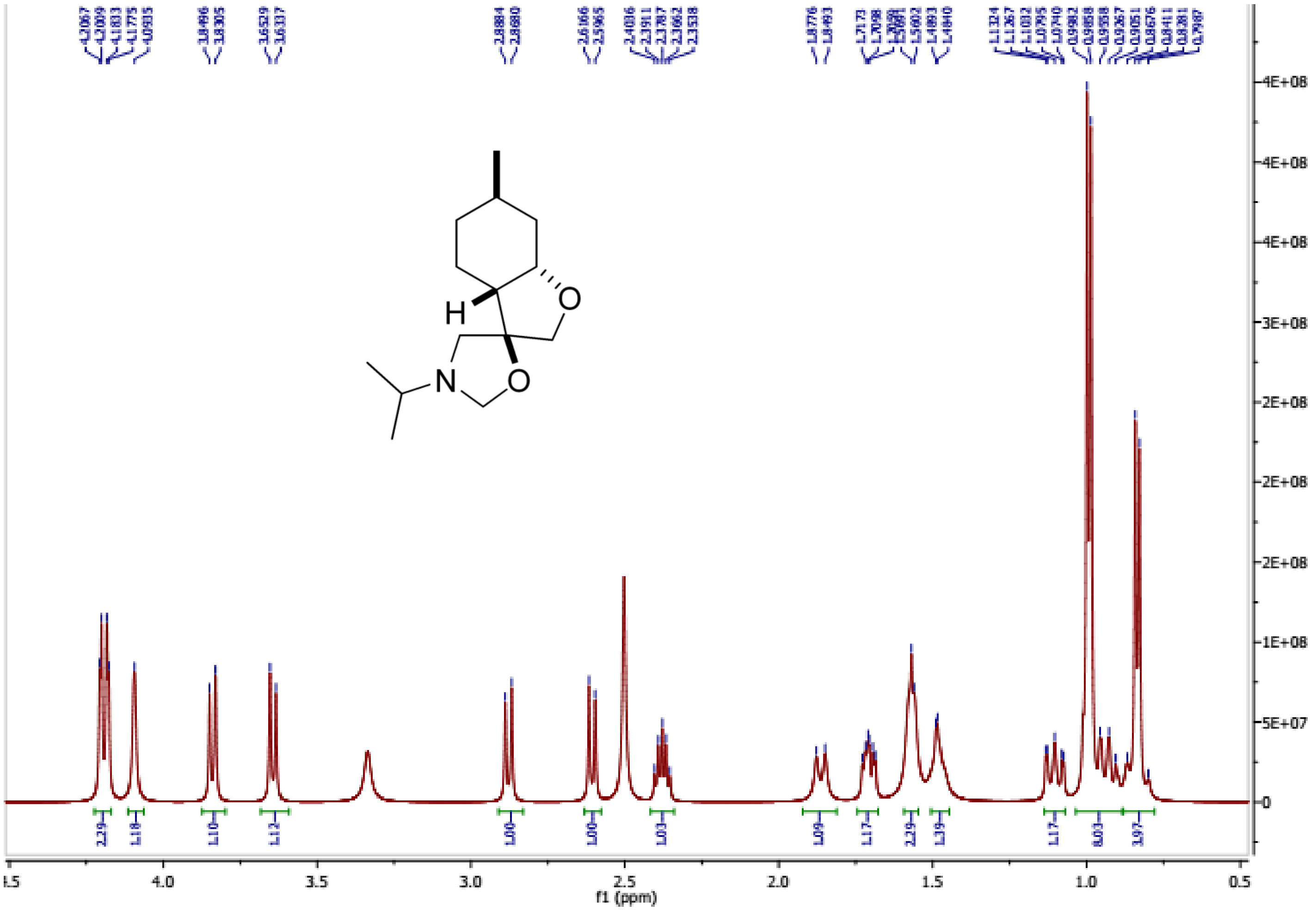

^{13}C-NMR of compound **15**

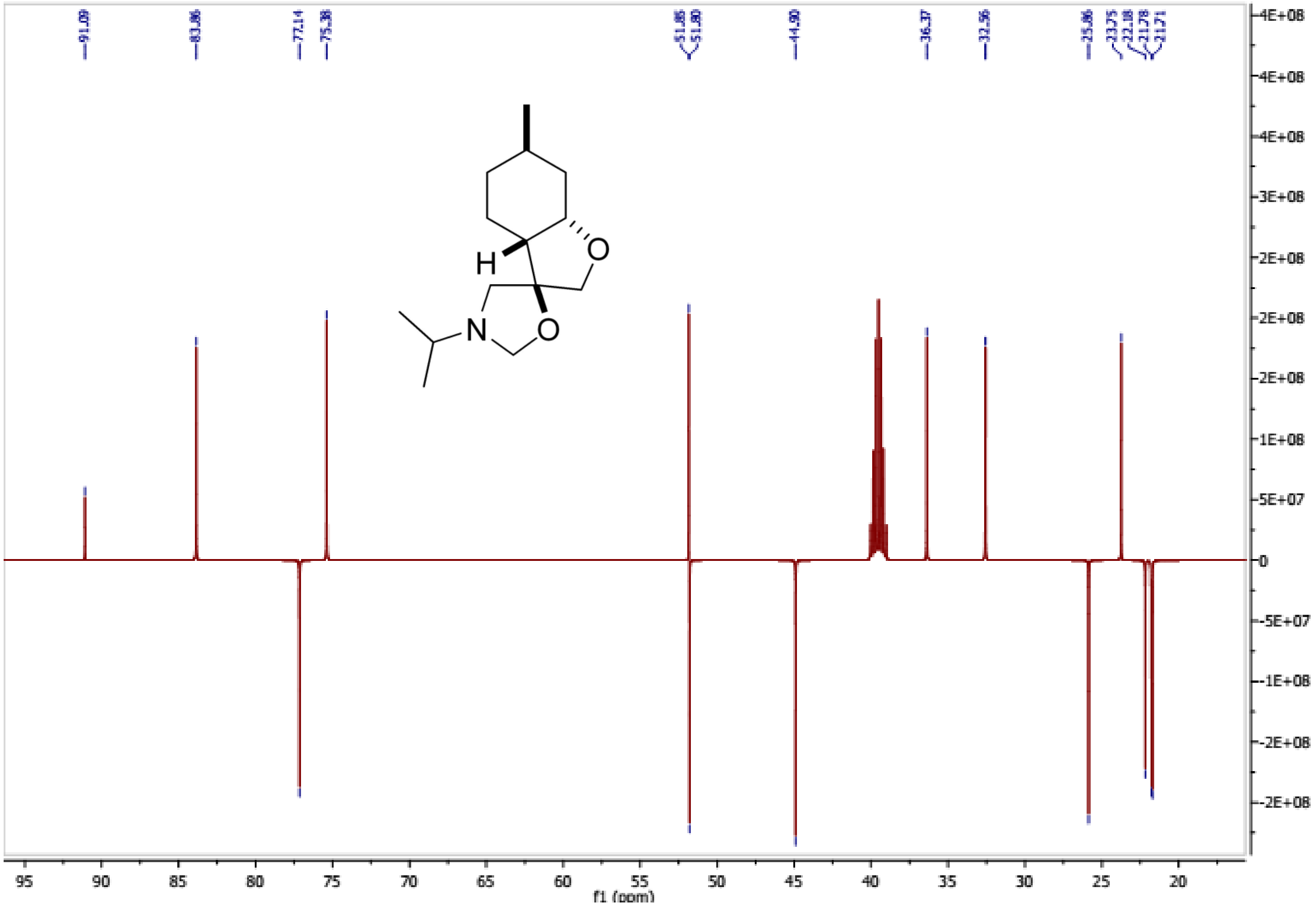

Supplement: Supplementary file 1 [file molecules-25-00021-s001.pdf]
